# Supplementary material for: Voltage gating and 4-aminopyridine inhibition in the Shaker Kv channel revealed by a closed-state model
Source: Biophys J. 2025 Jun 24;124(15):2500–10. doi: 10.1016/j.bpj.2025.06.029 (PMC12392982; doi:10.1016/j.bpj.2025.06.029)
Supplement: Document S1. Figures S1–S13 and Tables S1 and S2 [file mmc1.pdf]

Biophysical Journal, Volume *124*

Supplemental information

Voltage gating and 4-aminopyridine inhibition in the Shaker Kv channel revealed by a closed-state model

Bernardo I. Pinto-Anwandter

Supplementary information

Voltage Gating and 4-aminopyridine Inhibition in Shaker Kv Channel Revealed by Closed-State Model

Authors

Bernardo I Pinto-Anwandter [1*]

Affiliations

1 Department of Biochemistry and Molecular Biology, University of Chicago, Chicago, IL, 60637, USA

Correspondence

* Corresponding author, correspondence to pintobi@uchicago.edu

Supplementary Figures

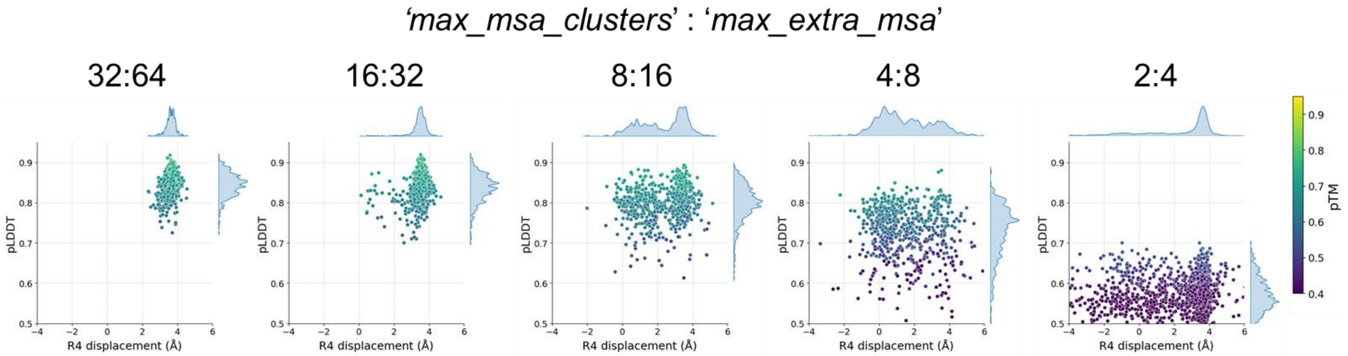

Supplementary figure 1: Conformational sampling parameter exploration for the Shaker VSD. Plot of R4 displacement vs pLDDT for AF2 generated models (600 models per plot) of Shaker VSD, using different '*max_msa_clusters*' and '*max_extra_msa*' parameters. Points colored according to pTM score. Side plots show the kernel density estimates distribution for each axis.

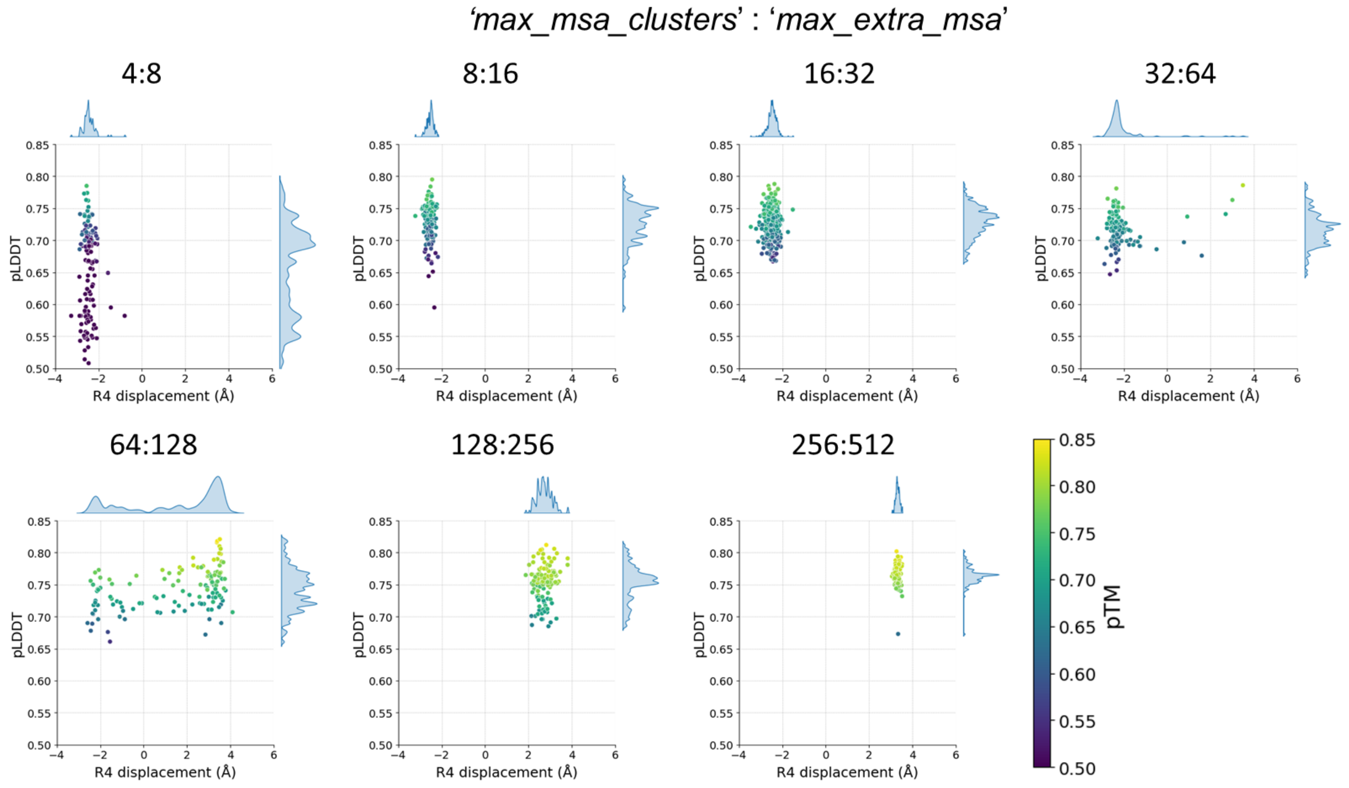

Supplementary figure 2: R4 displacement for the full channel using R4down template and different MSA subsampling parameters. Plot of R4 displacement vs pLDDT for AF2 generated models (128 models per plot) of Shaker tetrameric channel (residues 215-495) using R4down template using different MSA subsampling depth parameters. Points colored according to pTM. Side plots show the kernel density estimates distribution for each axis.

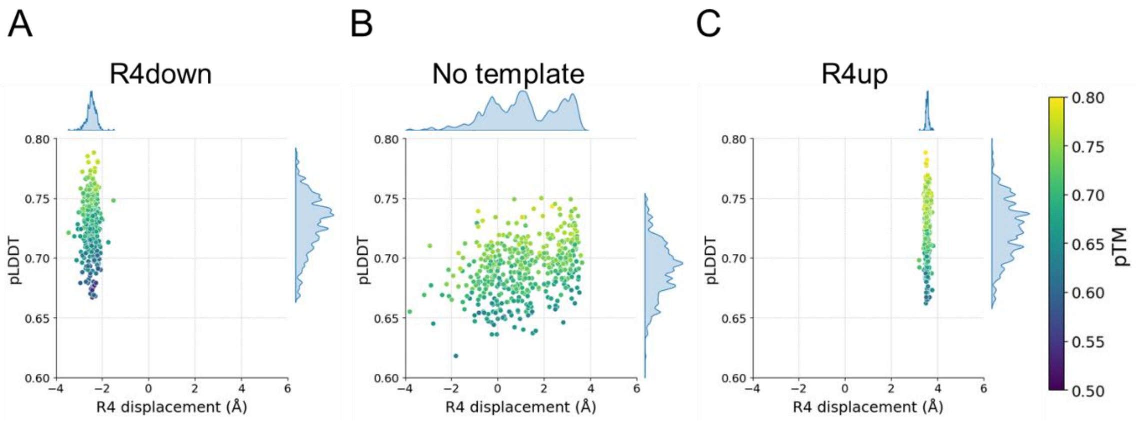

Supplementary figure 3: R4 displacement for the full channel with different VSD templates. Plot of R4 displacement vs pLDDT for AF2 generated models (400 models per plot) of Shaker tetrameric channel (residues 215-495) using R4down template (**A**), no template (**B**) or R4up template (**C**). MSA subsampling parameters used was 16:32. Points colored according to pTM. Side plots show the kernel density estimates distribution for each axis.

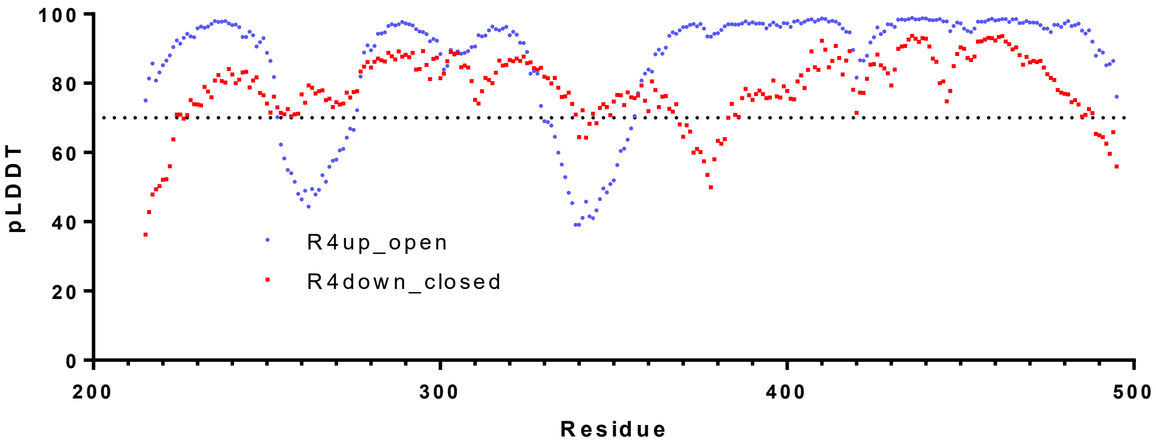

Supplementary figure 4: Per residue pLDDT metrics. Plot of the pLDDT values for each residue in the selected closed and open states. The dashed line indicates the cutoff values of 70 used to discriminate high pLDDT values.

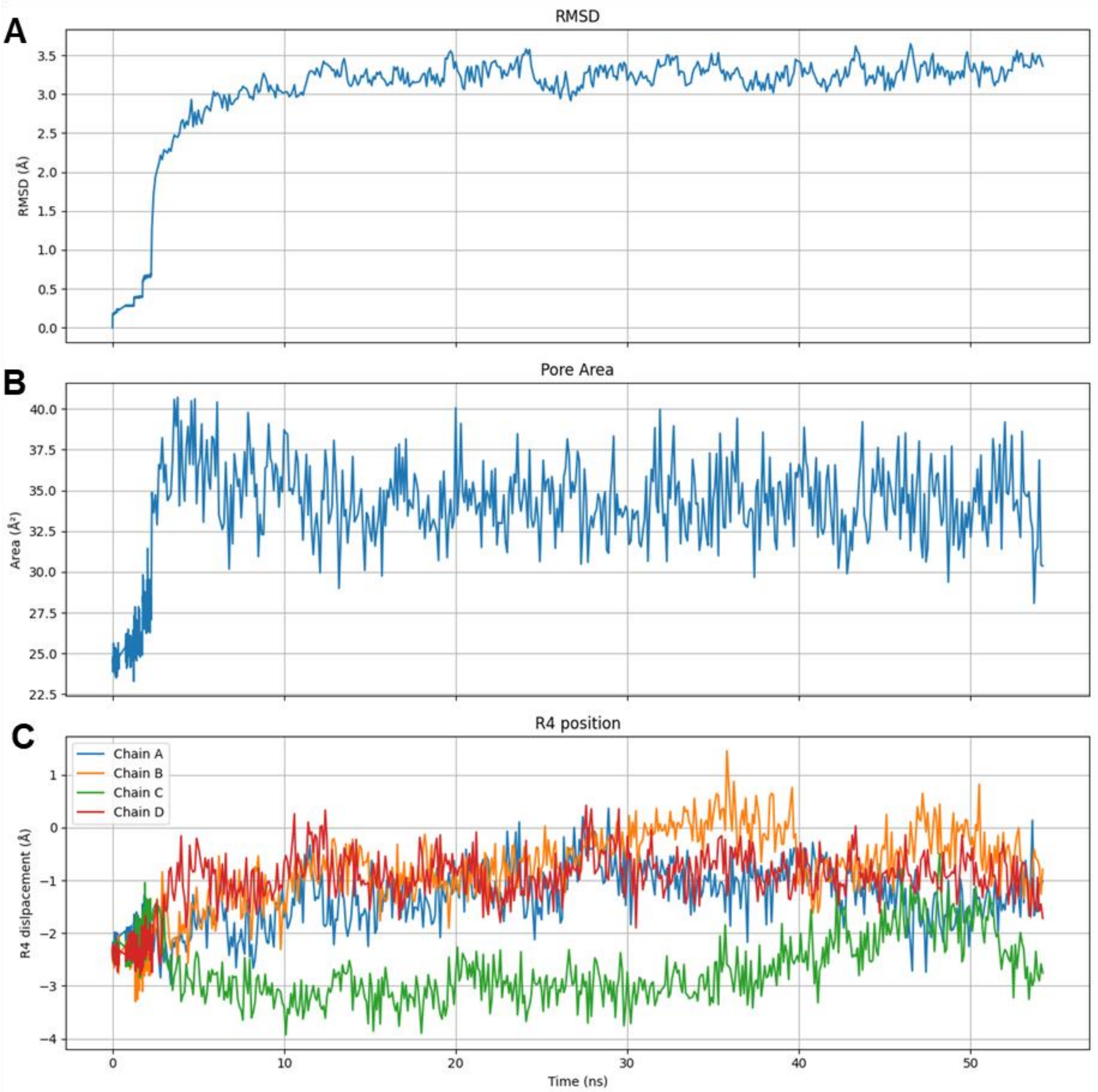

Supplementary figure 5: Analysis of molecular dynamics simulations of the closed state. **A)** Backbone RMSD, **B)** quadrilateral pore area at the level of V474 and **C**) R4 displacement for each VSD of the closed R4down model during a 55 ns MD simulation. The first 2 ns correspond to the equilibration of the system.

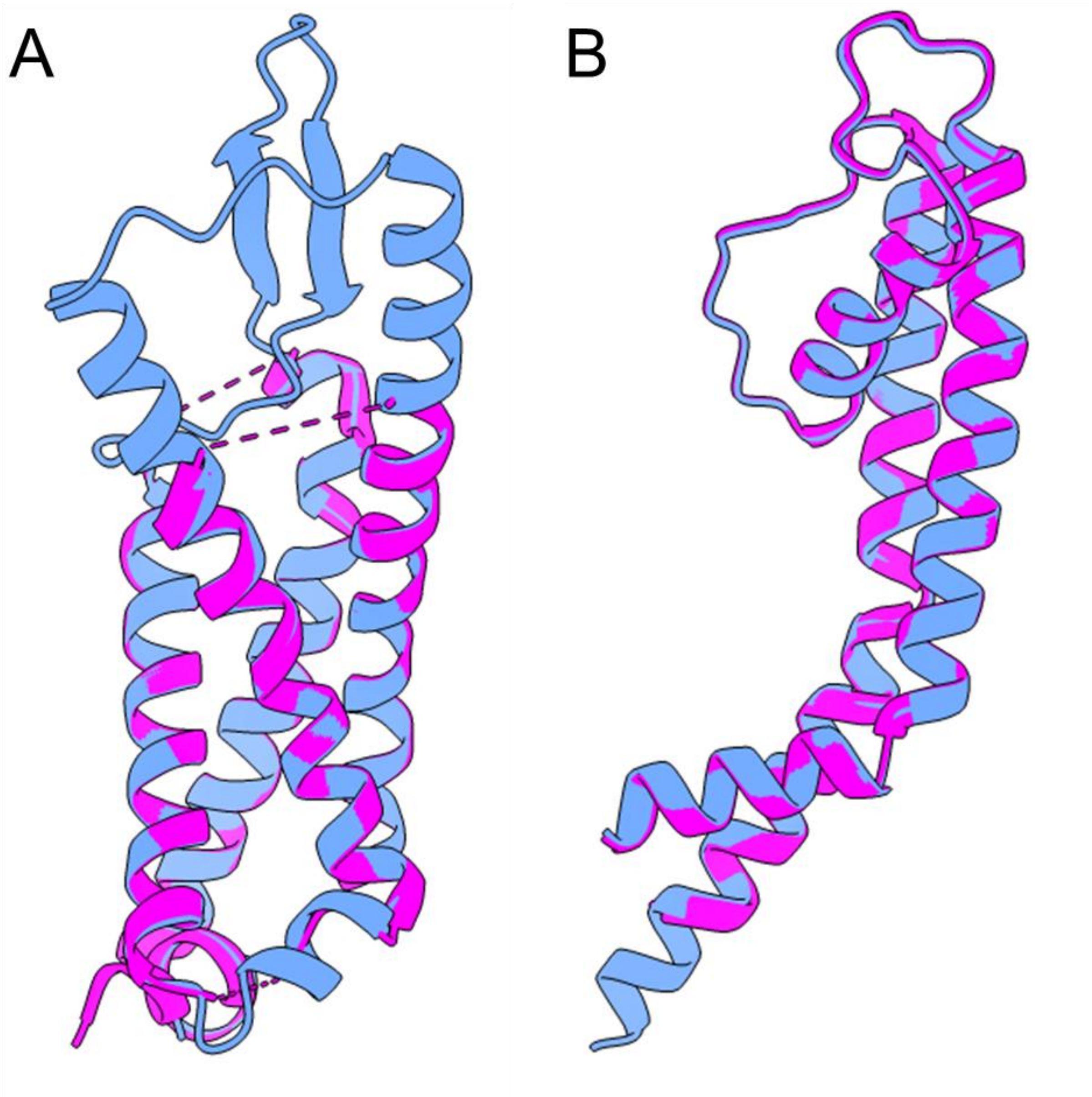

Supplementary figure 6: Comparison between R4up open model and structure of the WT channel. Aligned structures of the WT channel (PDB:7sip (22), magenta) and R4up open model (blue) for the VSD (**A**) and PD (**B**) of a single subunit (RMSD: 0.87 Å).

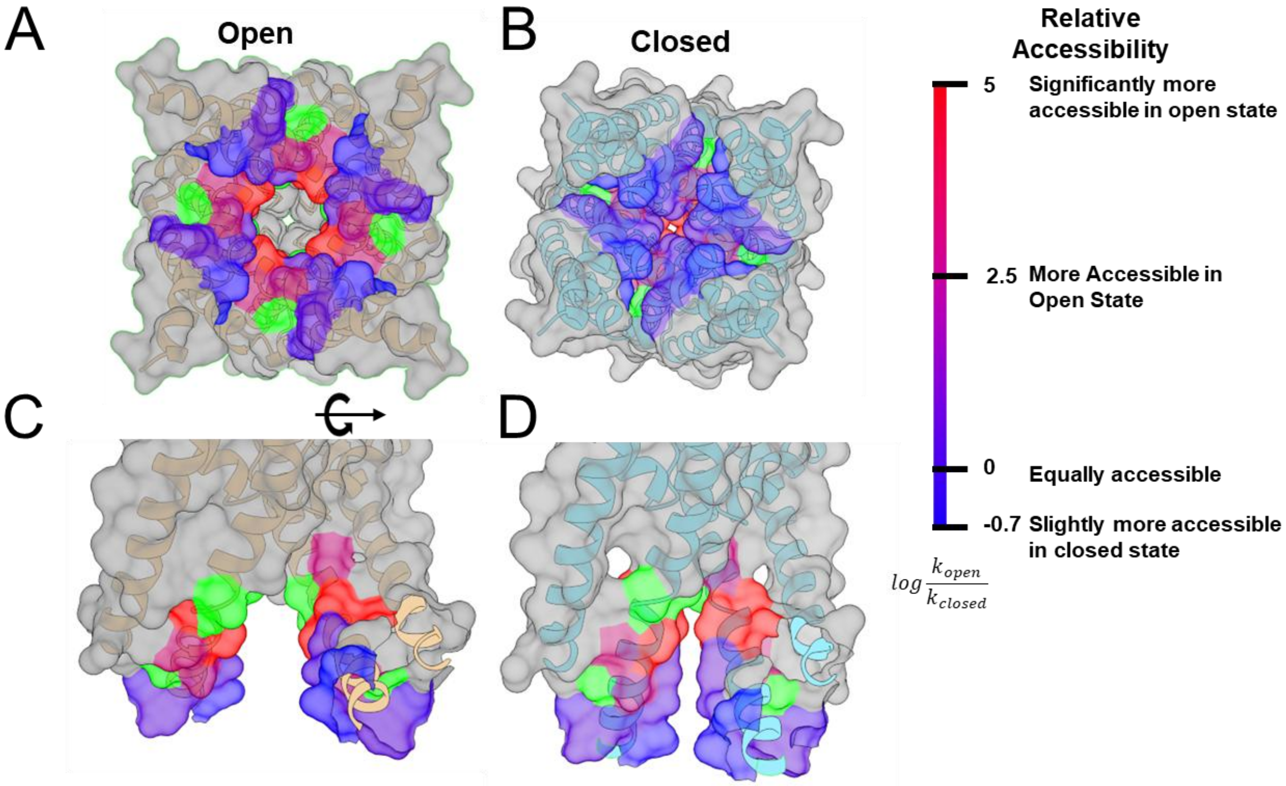

Supplementary figure 7: Mapping relative accessibility data into open and closed conformations. Surface representation of the PD of open (**A, C**) and closed (**B, D**) models colored according to experimental data of relative accessibility changes between open and closed states. Data adapted from ref (26), relative accessibility calculated as log (k_{open}/k_{closed}), when k_{closed} was unable to be measured (less than 1 M s^{-1}) value was set to 5. Green indicates residues that show no discernible effect upon application of modifying reagents. Gray indicates residues not tested.

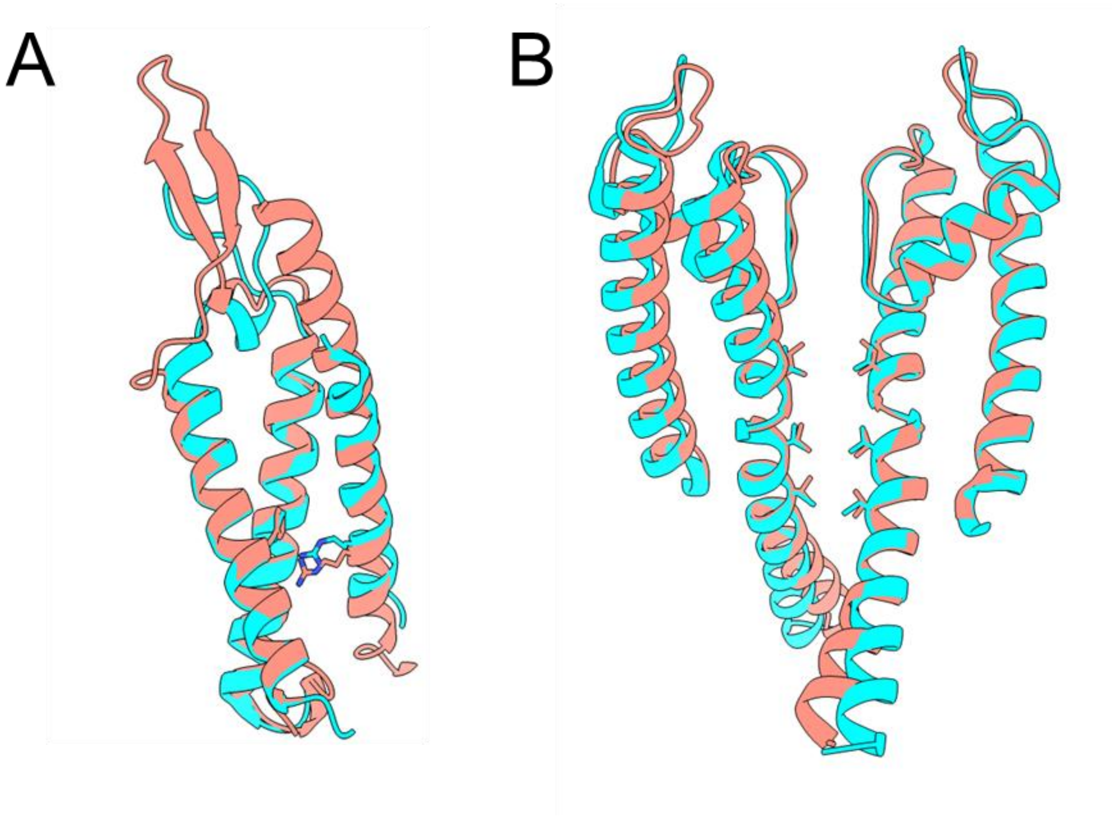

Supplementary figure 8: Comparison between R4down closed model and structure of putative resting Kv4.1 channel. Aligned structures of the putative resting Kv4.1 channel (PDB:7UKF (27), cyan) and R4up closed model (orange) for the VSD (S3 removed) of a single subunit (**A**) and PD of opposed subunits (**B**).

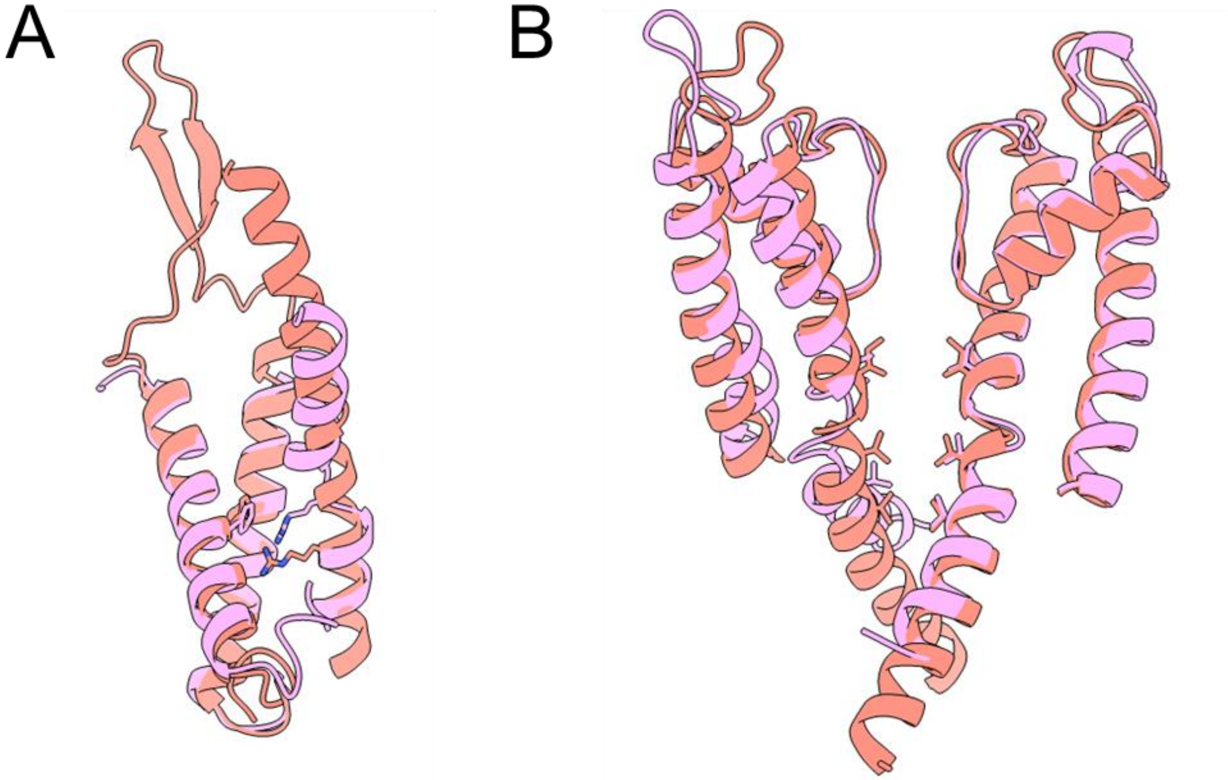

Supplementary figure 9: Comparison between R4down closed model and late deactivated state model from Jensen et al. (28)**.** Aligned late deactivated model of the Kv1.2/2.1 chimera (pink) and R4down closed model (orange) for the VSD of a single subunit (S3 removed) (**A**) and PD of opposed subunits (**B**).

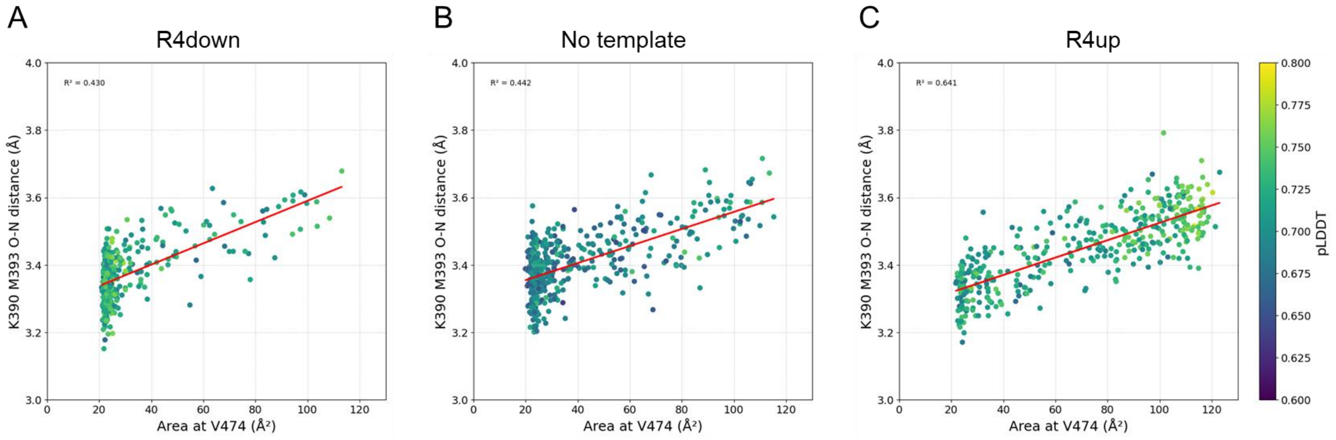

Supplementary figure 10: Relation between the area of the pore and the K390-M393 O-N distance for the full channel with different VSD templates. Plot of quadrilateral area at the level of V474 vs K390-393 O-N distance for AF2 generated models (400 models per plot) of Shaker tetrameric channel (residues 115-495) using R4down template (**A**), no template (**B**) or R4up template (**C**). MSA subsampling parameters used was 16:32. Points colored according to pLDDT. Red line shows linear fit.

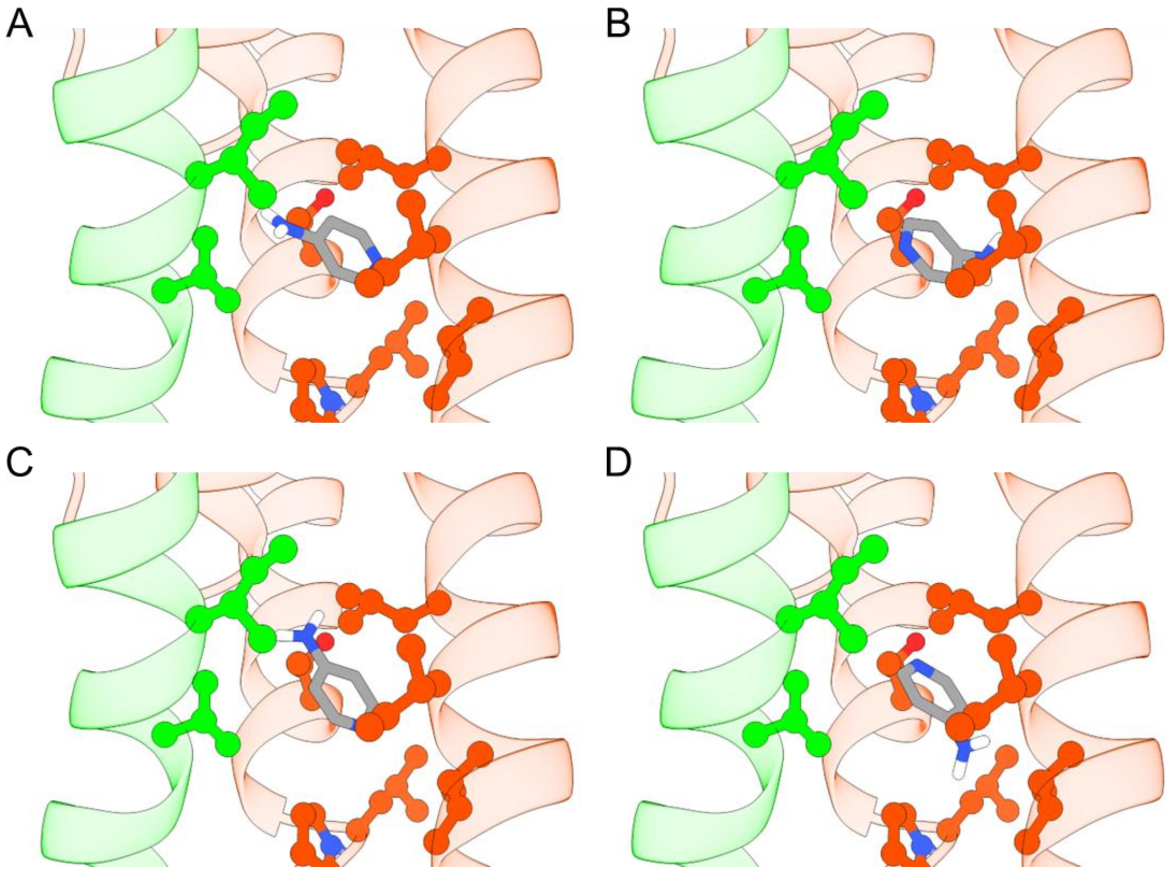

Supplementary figure 11: Docking poses and binding site of 4-AP. A-D) Representative structures of the four most favorable docking conformation identified. The protein is shown in cartoon representation with one subunit S5 and S6 in red and an adjacent subunit S5 in green, binding pocket residues are shown in stick representation. The predicted binding energies for each model are (**A**) -4.2, (**B**) -4, (**C**) -3.9 and (**D**) -3.8 kcal/mol.

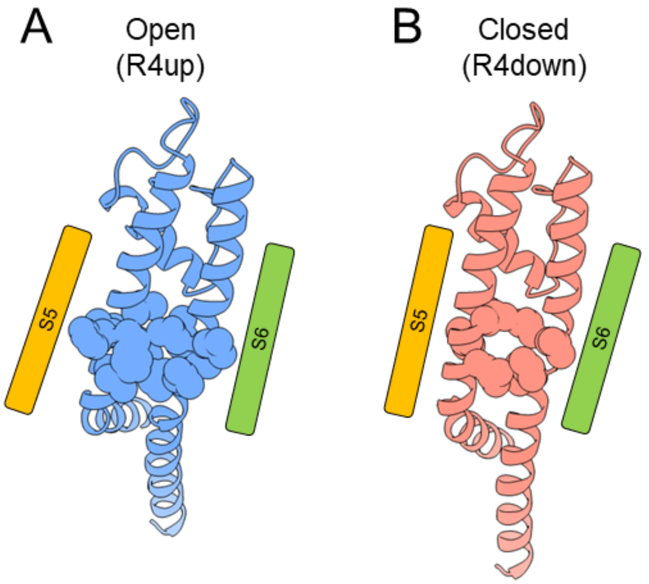

Supplementary figure 12: Formation of the 4-AP binding cavity on the closed state. Open (**A**) and closed (**B**) state models showing the PD region that forms the 4-AP binding cavity. Shown in sphere representation are the residues in S5 (398 to 403) and S6 (468 to 473) that form the cavity.

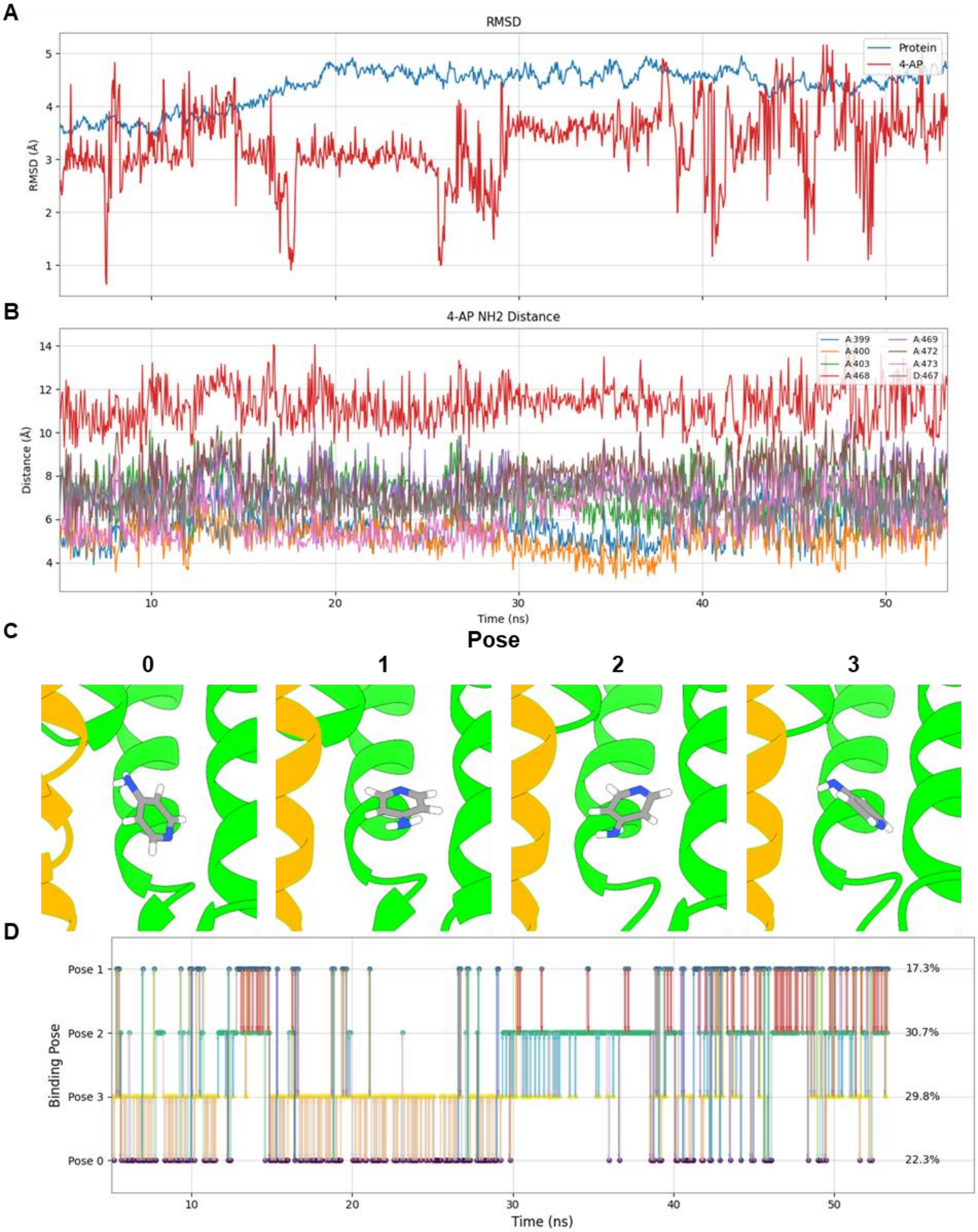

Supplementary Figure 13. Analysis of molecular dynamics simulations of the 4-AP bound state. A) Backbone RMSD of protein (blue) and 4-AP (red) over the 50 ns simulation. **B**) Time evolution of distances between the 4-AP NH_2 group and Cα atoms of the binding site. **C**) Representative structures of the four distinct binding poses (0-3) identified. The protein is shown in cartoon representation with one subunit in S5 and S6 in green and an adjacent subunit S5 in yellow, 4-AP is displayed in stick representation. D) Temporal distribution of the four binding

poses over the simulation Pose transitions are represented by vertical lines connecting the different states. Trajectories were analyzed after 5 ns.

Supplementary Table 1: GV fit parameters Shaker WT, ILT and 393 mutants

	V50 (mV)	z (e_0)
**WT**	-21.1 ± 0.5	2.6 ± 0.4
**M393A**	-26.25 ± 0.8	2.3 ± 0.7
**M393P**	-60 .1 ± 0.4	4.8 ± 0.4
**ILT**	133 ± 2	1.17 ± 0.06
**ILT-M393A**	107.3 ± 0.4	1.98 ± 0.05
**ILT-M393P**	0.4 ± 0.9	2.4 ± 0.2

*Fits were calculated using a two-state model (Eq. 1)

Supplementary Table 2: QV parameters for Shaker WT, ILT and 393 mutants

	z1 (e_0)	V1 (mV)	z2 (e_0)	V2 (mV)
**WT**	1.9 ± 0.3	-55 ± 2	3.5 ± 0.3	-34 ± 1
**M393A**	1.3 ± 0.2	-66 ± 3	3.3 ± 0.3	-46 ± 1
**M393P**	1.6 ± 0.3	-69 ± 4	4.5 ± 0.2	-72 ± 1
**ILT**	1.93 ± 0.06	-87.6 ± 0.5	1.5 ± 0.2	127 ± 4
**ILT-M393A**	1.9 ± 0.06	-92.5 ± 0.5	2.0 ± 0.1	104 ± 1
**ILT-M393P**	1 ± 0.1	-97 ± 9	0.5 ± 0.1	-76 ± 15

*Fits were calculated using a sequential three state model (Eq. 2) for WT, 393P, 393A and ILT_393P or fitting each component separately for ILT and ILT_393A (Eq. 1).
