# Supplementary material for: Voltage gating and 4-aminopyridine inhibition in the Shaker Kv channel revealed by a closed-state model
Source: Biophys J. 2025 Jun 24;124(15):2500–10. doi: 10.1016/j.bpj.2025.06.029 (PMC12392982; doi:10.1016/j.bpj.2025.06.029)
Supplement: Document S2. Article plus supporting material [file mmc6.pdf]

Biophysical Journal
Article

Biophysical Society

Voltage gating and 4-aminopyridine inhibition in the Shaker Kv channel revealed by a closed-state model

Bernardo I. Pinto-Anwandter[1,*]
[1]Department of Biochemistry and Molecular Biology, University of Chicago, Chicago, Illinois

ABSTRACT The generation and propagation of action potentials in neurons relies on the coordinated activation of voltage-dependent sodium and potassium channels. The Kv1 (Shaker) family of potassium channels drives the repolarization phase of the action potential by opening and closing their pore, a process controlled by a voltage sensor domain. However, a molecular description of how the voltage sensor domain drives pore gating has been constrained by a lack of closed-state structures. Here, we present a structural model of the closed Shaker channel that reveals the structural basis of voltage gating. Using AlphaFold2-based conformational sampling, we identified a partially activated state of the voltage sensor which, when modeled with the full channel, produced a closed state. Based on this model we demonstrate that breaking a backbone hydrogen bond between the S4-S5 linker and S5 helices is a critical part of the activation pathway. Docking studies revealed a hydrophobic cavity in the closed pore that binds 4-aminopyridine, a potassium channel inhibitor used to enhance nerve conduction in multiple sclerosis. Our results demonstrate how the voltage sensor movement drives pore opening and provide a structural framework for developing new therapeutic agents targeting the closed state. We anticipate that the novel methods used in this work will allow the characterization of conformational dynamics in voltage-gated ion channels, enabling drug design efforts focused on state-dependent modulation of ion channels for neurological disorders treatment.

SIGNIFICANCE Ion channels govern the electrical properties of cells. Among them, voltage-gated potassium channels Kv1 family are crucial for neuronal activity and represent important therapeutic targets for neurological disorders. Despite decades of structural and biophysical studies we still lack a molecular picture of how voltage drives the opening of the channel pore. Using AlphaFold2 we identified a structural model of the closed Shaker Kv1 potassium channel that reveals the mechanism by which the voltage sensor domain controls channel opening. Through docking analysis of the closed state, we also identified a key drug binding site.

INTRODUCTION

Voltage-gated potassium channels of the Shaker subfamily (Kv1) are widely expressed throughout the nervous system and in tissues such as the heart, vasculature, and immune system where they play a central role in cellular electrical signaling (1–4). These channels are composed of four subunits, each containing six transmembrane segments (S1-S6), where S1-S4 comprise the voltage sensor domain (VSD) and S5-S6 the pore domain (PD). Upon membrane depolarization the activation of the VSD drives the opening of the PD (5,6). Activation of the VSD is achieved by the translocation of charged arginine residues (R1-R4) in the S4 from an intracellularly exposed to an extracellularly exposed conformation (7–10). In this process, the translocation of R4 is the critical step that allows channel opening, and mutations that affects its translocation greatly shift the voltage dependence of activation (11,12). Despite extensive research into the gating mechanism of these channels, the precise details of how the VSD interacts with and controls the PD opening remain unclear. Our understanding of how VSD movement triggers pore opening and closure is limited in part because no structures of Kv1 channels in the closed state have been reported.

Their involvement in diverse physiological processes has made Kv1 channels attractive targets for therapeutic modulation; for instance, Kv1.1 and Kv1.2 have been investigated for epilepsy and pain management and Kv1.5 is a potential target for atrial fibrillation treatment due to its atrium-specific expression in the heart (3). A notable example of a Kv1 channel modulator that has shown therapeutic potential is dalfampridine also known as 4-aminopyridine (4-AP), a

Submitted February 3, 2025, and accepted for publication June 23, 2025.
*Correspondence: pintobi@uchicago.edu
Editor: Brad Rothberg.
https://doi.org/10.1016/j.bpj.2025.06.029
© 2025 The Author. Published by Elsevier Inc. on behalf of Biophysical Society.
This is an open access article under the CC BY license (http://creativecommons.org/licenses/by/4.0/).

nonselective Kv channel inhibitor. Dalfampridine has been found to improve electrical conduction in damaged nerve fibers by blocking Kv1.1 and Kv1.2 channels and has demonstrated particular promise in the treatment of multiple sclerosis (MS) (13). A slow-release formulation of dalfampridine showed significant improvements in walking ability for individuals with MS (14,15). This success led to its approval as a treatment option for MS patients, highlighting the potential of Kv1 channel modulators in addressing specific symptoms of neurological disorders. The development of selective Kv1 channel modulators may provide new therapeutic options with reduced side effects compared with less selective treatments, making Kv1 channels an important target for drug discovery efforts (3). Elucidating the closed state structure of Kv1 channels could provide mechanistic details about their gating and enable the design of selective and effective drugs that target specific conformational states.

The development of machine learning methods for protein structure prediction, such as AlphaFold2 (AF2) and RoseTTAFold, has revolutionized the field of structural biology by providing highly accurate predictions that often rival experimental methods in precision (16–18). These algorithms usually predict a single conformation, in contrast with our understanding of proteins as existing under a constant dynamic equilibrium. In the AF2 pipeline, a multiple sequence alignment (MSA) against the query sequence is generated and a random subset of this MSA is then used during the inference stage to predict the structure of the protein. Modification of the size of this MSA subset (MSA subsampling) allows the system to explore different conformations (19). This MSA subsampling method has been applied to obtain different conformations of transporters, kinases, and GPCRs (19–21). We used MSA subsampling to explore the conformational diversity of the VSD in the prototypical Shaker Kv1 channel. This led to the prediction of a novel conformation in which the fourth (most intracellular) sensing arginine (R4) of the VSD has not translocated to the extracellular side. We used this novel conformation (R4down) of the VSD as a template to predict the closed state of the PD of the tetrameric channel. The R4down closed model suggests an electromechanical coupling mechanism between the VSD and PD, in which the VSD pushes into the pore to close it. This closure is driven by interactions between the S4-S5 linker and the C-terminus of the S6 segment that lead to a translation and rotation of S6 that finally occludes the permeation pathway. From the predicted structure and electrophysiology analysis of mutants, we identified the region between the S4-S5 linker and S5 as a critical pivot point where the breakage of a backbone hydrogen bond plays a central role in the activation process. Through docking, molecular dynamics simulations, and electrophysiological experiments, we identified a cavity where 4-AP binds. This cavity formed by S5 and S6 in the closed state is absent in the open state, supporting a closed state stabilization mechanism for 4-AP inhibition. These findings offer a detailed picture of the structural dynamics of voltage gating and provide a foundation for rational drug design against the closed state of Kv1 channels.

RESULTS

Conformational sampling of the VSD

To study the conformational dynamics of the VSD we used the *Drosophila* Shaker channel since it has been extensively characterized and its structure was released after AF2 training, preventing training data bias (18,22). To obtain different conformations of the Shaker channel VSD (residues 224–382; monomer) we used MSA subsampling (16,19). During our initial screening run we generated models with different levels of MSA subsampling (∼600 each). To analyze the changes on the VSD, we calculated the displacement of the fourth arginine (R4) from the intracellular to extracellularly exposed conformation, defined by the F290 residue. This residue is critical for the gating of the Shaker channel as it forms part of the hydrophobic plug through which the arginine residues of the S4 translocate to the extracellular side during channel opening (11,12,23). Critically F290 modulates the translocation of R4, thus it is expected that a channel in which R4 is found below (or more intracellular than) F290 will be closed (12). We observed that decreasing the number of sequences resulted in the sampling of conformations with R4 displaced into the intracellular side (Fig. S1). The predicted template modeling and predicted local distance difference test (pLDDT) scores, which correspond to confidence metrics of the model, are also reduced with decreased MSA subsampling (higher score is better). To balance the need for models with R4 displaced and good confidence scores we generated 6000 models using MSA subsampling parameters that show a wide array of R4 displacements while still maintaining high confidence scores (Fig. 1 *A*). The model with the maximum predicted template modeling and pLDDT score and R4 displacement below F290 was selected. When compared against the wild-type (WT) VSD experimental structure (22) we can observe that the S4 helix shows a displacement of ∼6 Å into the intracellular side and that R4 is found below the F290 residue (Fig. 1 *B*). We can then use this R4down conformation to model the closed state of the channel.

A model for the closed state of the Shaker channel

We used the R4down model to predict the closed channel structure (residues 115–495; tetramer), employing the W434F mutation to minimize selectivity filter distortions observed with the WT sequence. When AF2 uses templates, it is possible that the template information will not be reflected in the final structure because it is overridden by the

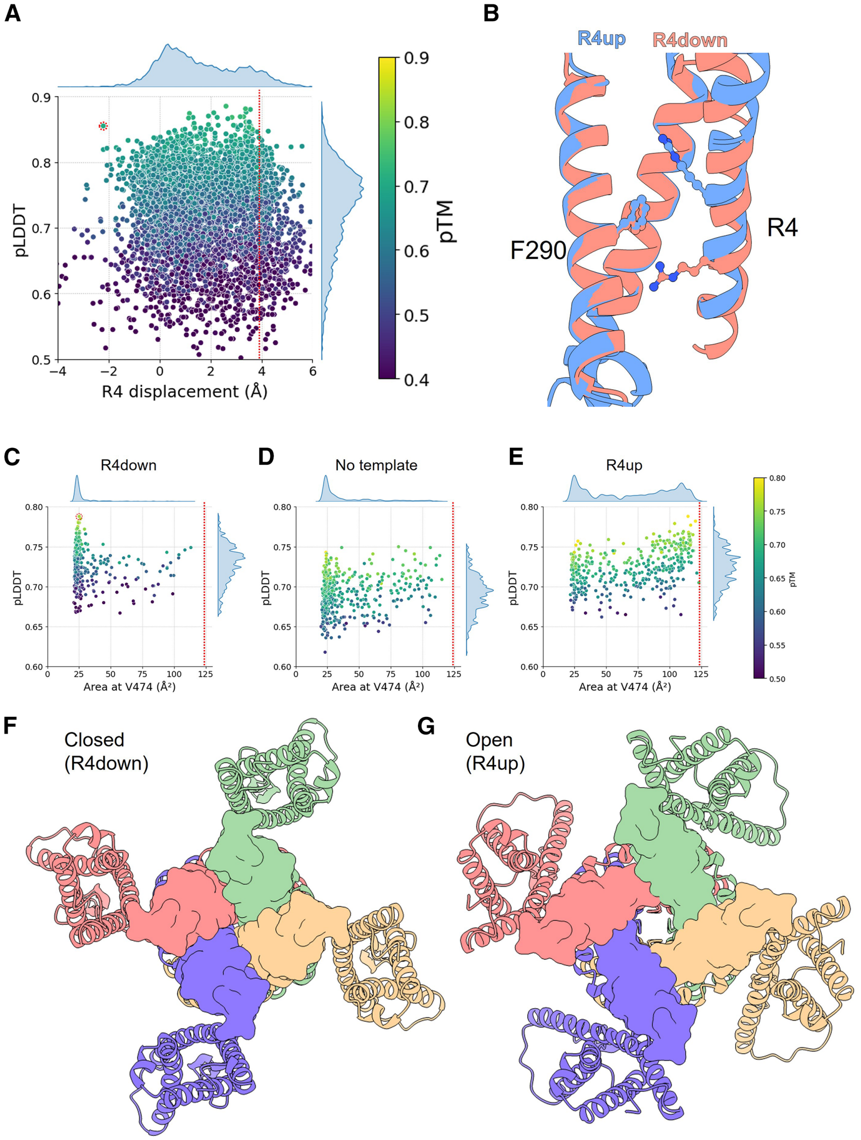

FIGURE 1 Obtaining a closed state model for the Shaker channel. (*A*) Plot of R4 α carbon displacement versus pLDDT for AF2 generated models of Shaker VSD (6000 models). Points colored according to the predicted template modeling (pTM) score. MSA subsampling parameters used was 4:8 (“max_seq”:“max_extra_seq”). Circle indicates the selected VSD model, dotted red line indicates the displacement in the WT structure. (*B*) Comparison between the VSD from the Shaker WT structure (R4up, PDB: 7sip (22)) and the selected VSD model (R4down), showing position of residues F290 and R4. S3 was removed for clarity. (*C–E*) Plot of quadrilateral area at the level of V474 versus pLDDT for AF2 generated models (400 models per plot) of Shaker tetrameric channel (residues 215–495) using R4down template (*C*), no template (*D*), or R4up template (*E*). Dotted line indicates the area in the WT structure. MSA subsampling parameters used was 16:32. Circle in (*C*) indicates the selected model. Side plots show the kernel density estimates distribution for each axis. (*F* and *G*) Intracellular view of the tetrameric Shaker channel in the closed (R4down) (*F*) and open R4up (*G*) conformations, each subunit is shown in a different color. Surface representation is shown for residues of the S6 C-terminal region.

information of the MSA (19,24). To test this effect, we used different MSA subsampling parameters with the R4down model as a template (Fig. S2). We observe that, as more sequences are included, the final structure reverts the movement of R4 to the extracellular facing (“up”) configuration. Thus, we used MSA subsampling parameters in which the resulting models maintain the R4down conformation. To quantify the opening of the PD in the different models we calculated the quadrilateral area of the pore at the level of valine 474, since this member of the conserved proline-valine-proline (PVP) hinge motif shows large changes in accessibility between open and closed states (25). We found that incorporating the R4down template leads to the modeling of a closed channel with high-confidence metrics compared with models without templates or using the WT structure VSD as a template (R4up) (22) (Fig. 1 *C–E*). Most of the models with the R4down template have areas of less than 30 $Å^2$ and many of them have high-confidence metrics (pLDDT > 0.75). On the other hand, the models without a template do not have high-confidence metrics, despite having conformations lower than 30 Å and exploring different R4 displacement (Fig. S3 *A* and *B*). Finally, the models obtained using the R4up template show consistent higher-confidence values for higher-area values (Spearman correlation = 0.47), and in all these models R4 is found above (or more extracellularly than) F290 (Fig. S3 *C*). For further analysis we selected the closed state model with the highest pLDDT score (*circle* in Fig. 1 *C*). This model has high pLDDT values (>70) across the different residues, dipping into lower values at the beginning of S1 (residues 215–226), S3-S4 linker (residues 340–345), the end of the S4 beginning of the S4-S5 linker (residues 369–382), and

the end of S6 (residues 489–495) (Fig. S4). These low pLDDT regions do not preclude our analysis of the rest of the model. To further validate the stability of the proposed closed channel conformation, molecular dynamics simulations were performed. During a 50 ns simulation, the channel maintains the R4down position, the closed gate, and its overall structure with a final backbone root mean-square deviation of about 3.5 Å (Fig. S5).

We compared the closed state model against a model using the R4up template that is similar to the WT structure with the additional benefits of having the loops modeled and extending the S6 region missing in the structure (Figs. S4 and S6). When looking at the R4down model from the intracellular side toward the pore we can clearly see an occlusion of the conduction pathway by the S6 helix, not seen in the R4up model (Fig. 1 *F* and *G*) indicating that this is a bona fide closed state (R4down and pore closed). We find several key differences between the open and closed models that provide a clear interpretation of the gating mechanism. In the closed R4down model the intracellular movement of the VSD generates a displacement of the S4-S5 linker and this movement of the S4-S5 resembles a lever with a pivot at the S5 helix (Fig. 2 *A*). This movement of the S4-S5 linker also induces a displacement of the C-terminus of the S6 helix, which produces a translation and rotation of the S6 that closes the channel (Fig. 2 *B* and *C*; Videos S1 and S2). The model shows residues I470, V474, and V478 as the residues that occlude the pore, thus forming the gate of the channel (Fig. 2 *D*; Videos S3 and S4). This mechanism fits well with previously published observations of accessibility changes in the S6 region during gating (Fig. S7) (25) and is remarkably similar to the proposed resting state structure of the Kv4.1 channel (Fig. S8) (26). We also compared this model with a late deactivated model of the Kv1.2/2.1 chimera obtained by long molecular dynamics simulations (27), where we also see that only one arginine has translocated into an intracellularly exposed conformation (Fig. S9). In this case we see a similar movement of the S4-S5 linker and gate for one subunit, despite differences in the position of the S4 indicating that the AF2 structural model can capture similar gating transitions as those derived from molecular dynamics simulations.

Methionine 393 acts as a pivot point for the S4-S5 linker movement during opening

When comparing the closed and open state models we observe that, when the channel closes, there is a movement of the S4-S5 linker that would allow lysine 390 (K390) carbonyl oxygen to form a backbone hydrogen bond with the amide nitrogen in methionine 393 (M393) of the S5 segment (Fig. 3 *A* and *B*). When assessing the distance between these atoms in all the models generated, we find that there is a linear relationship between the area of the pore and the distance between these two atoms (O-N distance), suggesting a critical role for hydrogen bond formation in the closing of the PD (Fig. S10). Based on this, we hypothesize that, if we prevent the carbonyl of K390 from interacting with the amide of M393, we can stabilize the open state of the channel. To achieve this, we introduced a proline at position 393 (M393P), which lacks the hydrogen necessary for the interaction with the carbonyl group of K390. We characterized the effect of this mutation by measuring the ionic currents in response voltage pulses and determining the steady-state conductance versus voltage curve (G-V). We find that the steady-state conductance of the M393P mutant has a −40 mV shift when compared with the WT channel, indicating a stabilization of the open state (Fig. 3 *C*; Table S1). To prevent confounding effects arising from

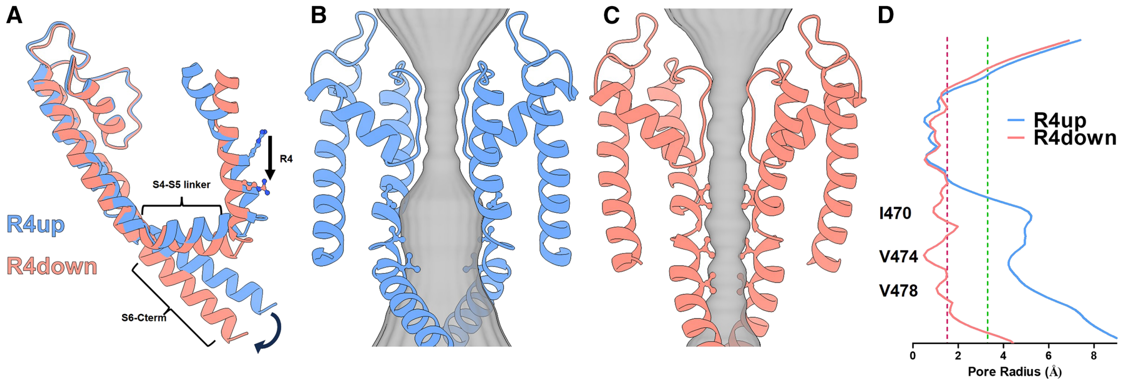

FIGURE 2 Conformational changes from open to closed state of Shaker. (*A*) Superposition of the S4 to S6 region of a single subunit in the open (R4up, *blue*) and closed (R4down, *red*) states of the Shaker channel. Arrows indicate two major conformational changes between states: the movement of the S4 helix (shown as the displacement of R4) and the S6 C-terminus. (*B* and *C*) Intramembrane view of two opposed subunits of the R4up open state (*B*) and R4down closed state (*C*). Residues I470, V474, and V478 side chains are shown in ball and stick representation. The pore diameter obtained through HOLE calculations is shown in gray. (*D*) Pore radius analysis for the open (*blue line*) and closed (*red line*) states. The *y* axis represents the position along the pore axis aligned with the structures in (*B*) and (*C*). The position of key residues I470, V474, and V478 are indicated, highlighting the region of major constriction in the closed state. Dashed red and green lines indicate radii of 1.5 and 3.3 Å corresponding to the ionic and hydrated radius of potassium, respectively.

modification of the side chain we also analyzed the effects of an alanine mutation at the 393 position (M393A) and found only a modest −5 mV shift. To further characterize the effects of these mutations we analyzed the VSD movement directly by measuring the gating currents using the nonconducting W434F mutant channel (28). Consistent with the G-V results, the charge versus voltage (Q-V) curve shows a leftward shift for M393P of −30 mV, while M393A was only −10 mV when compared against the WT (Fig. 3 *D*; Table S2).

Our model shows that the extracellular displacement of R4 is the event that breaks the K390-M393 hydrogen bond, therefore the proline mutations should affect primarily the last transition in the VSD activation, that is, the translocation of R4. To test this idea directly, we introduced the V369I, I372L, and S376T (ILT) mutations. The ILT mutant produces a drastic change in the G-V curve of the channel (half activation voltage, $V_{1/2}$ of +137 mV, Table S1), caused by the displacement of the last charge translocation step. This is observed as a split in the Q-V where about ∼10% of the charge appears at largely depolarized potentials, while the rest of the Q-V curve remains largely unchanged (12,29). We expect from the proposed effect of M393P on the last transition, that this mutation would counter the effects of ILT. Indeed, the ILT-393P mutant shows an activation $V_{1/2}$ of 0 mV and the Q-V curve develops without a clear split (Fig. 3 *E* and *F*). In contrast, ILT-393A maintains a highly depolarized activation voltage ($V_{1/2}$ = 107 mV) and a split Q-V curve. These results demonstrate that the M393P mutation, which prevents the H-bond formation, modifies the activation energy of the last charge translocation in a manner consistent with the R4down closed state conformation, providing experimental support for the closed model.

Identification of the 4-AP binding pocket

4-AP inhibits the final transition of the voltage sensor, which is essential for channel opening, much like the ILT mutations (30,31). In the presence of 4-AP, the last gating component is absent in the ILT mutant (12), suggesting that 4-AP further suppresses this transition, preventing the complete activation of the VSD. By targeting this transition, 4-AP effectively prevents the channel from reaching the open state, thereby inhibiting its function. Given its ability to stabilize this pre-open

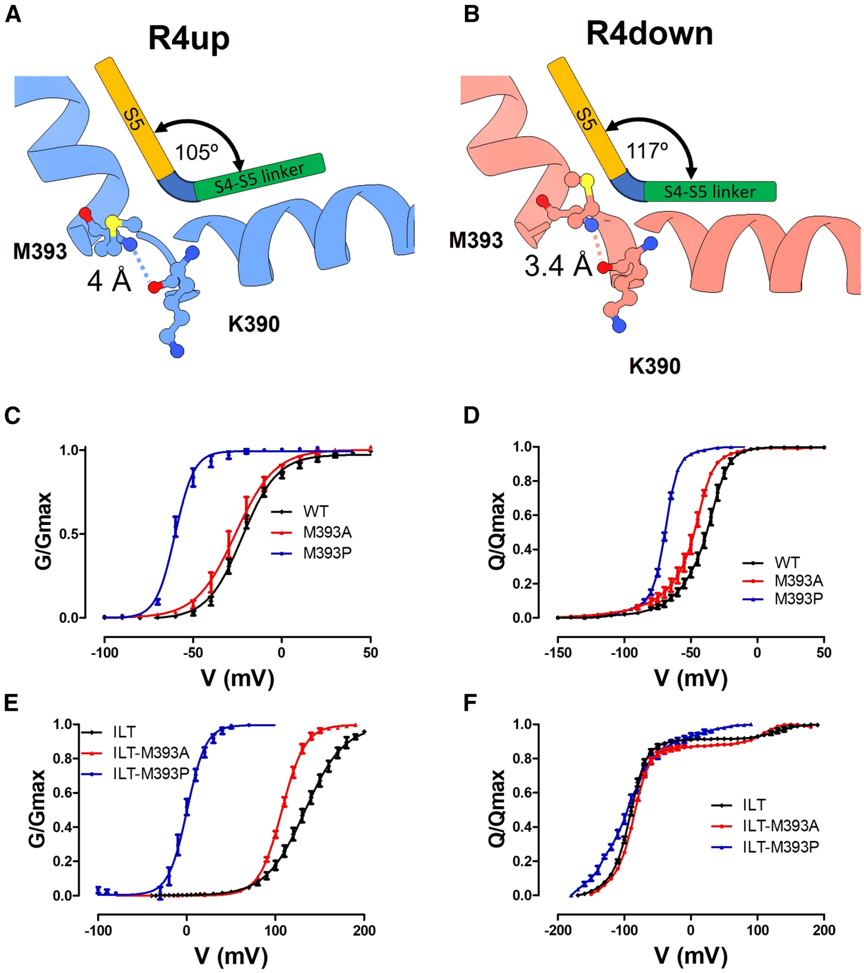

FIGURE 3 S4-S5 linker movement during opening involves breakage of a backbone hydrogen bond. (*A* and *B*) S4-S5 linker position relative to the S5 in the R4up open conformation (*A*) and the R4down closed conformation (*B*). The dashed line shows the distance between K390 carbonyl group and M393 amide group. The cartoon exemplifies the movement of the S4-S5 linker relative to the S5. (*C*) G-V relationship for the WT channel (*black*), M393A (*red*), and M393P (*blue*) mutants. (*D*) Q-V relationship for the WT channel (*black*), M393A (*red*), and M393P (*blue*) mutants. (*E*) G-V relationship for ILT (*black*), ILT-M393A (*red*), and ILT-M393P (*blue*) mutants. (*F*) Q-V relationship for ILT (*black*), ILT-M393A (*red*), and ILT-M393P (*blue*) mutants. Results shown as mean ± SEM, $n \geq 4$. Continuous lines correspond to a two-state fit (Eq. 1) in the GV curves and an interpolation between points in the Q-V curves.

state of Kv1 channels, 4-AP is an ideal candidate for docking analysis to identify its binding site in the R4down closed model. We performed docking analysis of 4-AP against the PD of the R4down closed model using AutoDock Vina (32,33). The docking analysis identified a hydrophobic cavity formed by residues in the S5 (L399, I400, L403) and S6 (V467, L468, T469, L472, P473) where 4-AP binds (Figs. 4 *A* and S11). This is consistent with previous reports of 4-AP affinity transplantation between Kv2.1 and Kv3.1 by exchange of the S5 and S6 regions, equivalent to residues 395–401 and 469–476 in Shaker (34). This cavity is absent in the open state of the channel, explaining the closed state stabilization effect of 4-AP (Fig. S12). Molecular dynamics simulations with bound 4-AP indicate that this binding is stable and that the 4-AP molecule has different possible stable conformations within the pocket (Fig. S13). We tested the inhibition by 4-AP in two mutants in this binding pocket L399A and V467I with the intention of modifying the cavity size. Upon application of 0.4 mM 4-AP the WT channel shows an inhibition of about 60% of the ionic current, while in L399A the current is almost eliminated and in V467I the current is marginally affected (Fig. 4 *B*). These results are explained by the changes in affinity produced by these mutations, the half-inhibitory concentration of 4-AP for L399A and V467I has a fivefold decrease and an estimated ∼100-fold increase, respectively, when compared with the WT (Fig. 4 *C*).

DISCUSSION

Using AF2 with MSA subsampling we obtained a closed state model of the Shaker Kv channel in which R4 has not translocated to the extracellular side. This intermediate represents a key step in the gating process, the last step before channel opening, distinct from the resting conformations observed in structures of other voltage-gated ion channels such as EAG1, Nav, and TPC channels, where typically two or more charges are displaced (35–37). The identified intermediate thus offers a unique snapshot of the voltage sensor in the transition between open and closed states. This model agrees with previous experimental observations of accessibility differences between closed and open channels and our mutational analysis provide experimental support for this model.

A proposed conserved mechanism for domain-swapped Kv channel activation

In domain-swapped Kv channels (Kv1-9), the VSD of one subunit interacts with the PD of an adjacent subunit rather than its own (38). This architectural distinction likely results in different mechanisms of electromechanical coupling during activation and deactivation.

Based on our modeling we propose a mechanism for the gating of the Shaker channel by which the movement of the

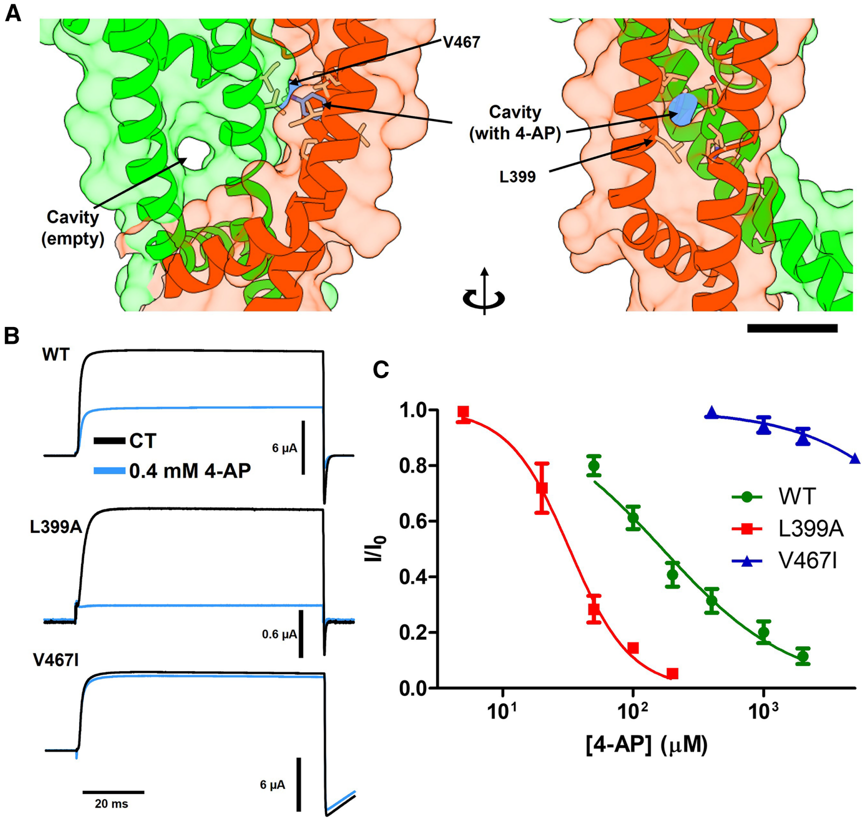

FIGURE 4 Identification of the 4-aminopyridine binding pocket. (*A*) Docking analysis results showing the hydrophobic cavity where 4-AP binds, formed by residues in the S5 (L399, I400, L403) and S6 (V467, T469, L472, P473). Scale bar, 1 nm. (*B*) Inhibition of ionic currents by 0.4 mM 4-AP in WT, L399A, and V467I mutant channels. (*C*) Dose-response curves for the WT channel (*green circles*, half inhibitory concentration $[IC_{50}]$ = 170 μM), L399A (*red squares*, IC_{50} = 32 μM), and V467I (*blue triangles*, IC_{50} = 28 mM). Results shown as mean ± SE, *n* ≥ 4.

voltage sensor regulates the opening of the pore. The VSDs undergo conformational changes in several steps in response to membrane depolarization. The last step shifts the most intracellular gating charge (R4) from the R4down (resting) state to the R4up (active) state. This movement exerts mechanical force on the S4-S5 linker, which in turn triggers a rotation and translation of the S6 helices, leading to the opening of the channel pore (Fig. 5; Videos S1, S2, S3, and S4). The model also indicates that the closed state is stabilized by a hydrogen bond between the S4-S5 linker and the S5 segment, suggesting that to open the channel work must be done by the VSD to disrupt this interaction. The concerted movement of the S4-S5 linker and S6 C-terminus explains why the complementary interaction between these regions is required for proper voltage gating and why mutations along the S4-S5 linker and S6 terminus interface produce different levels of VSD-PD uncoupling (39–42). The concerted movement of the S4-S5 linker and S6, and the subsequent expansion of the pore observed in this work is remarkably similar to the proposed activation mechanism for Kv4.1 (26). These results also contrast with the proposed mechanism of Nav channels (36), where the pore is constricted by a lateral pinching rather than a constriction mediated by movement between the S4-S5 linker and S6. Thus, our results suggest a common mechanism of voltage activation for domain-swapped Kv channels.

Explaining the foot in the door effect of quaternary ammonium derivatives

The structural change that opens the pore not only allows ion permeation but also creates an aqueous internal cavity lined by the S6 helices. This cavity becomes accessible to intracellular molecules, including quaternary ammonium (QA) derivatives such as tetraethylammonium. QA binds in the intracellular side of the selectivity filter, occupying the aqueous internal cavity formed by the S6 helices and blocking the channel (43,44). This blockage requires the activation gate to be open, and because the QA needs to be dislodged from its site to close the channel this block produces a slowing down of the closure kinetics, a mechanism aptly named "foot in the door" (45,46). When I470 is mutated to alanine or cysteine, the channel can close without the need to dislodge QA, which effectively traps the molecule in its site (47,48). In the closed state model, the internal cavity is constricted by residue I470 as the S6 rotates from the open to closed state. As a result, a QA molecule bound in the open state cannot remain in place during closure and must dissociate before the gate can close. Thus, when I470 is mutated to a smaller residue such as alanine or cysteine, the cavity no longer collapses completely, allowing the QA to remain bound and become trapped within the cavity.

The binding site of 4-AP and its inhibition mechanism

4-AP is a nonselective K^+ channel inhibitor used in the symptomatic treatment of MS (13). Thus, elucidating the inhibition mechanism of 4-AP could help in the development of novel selective and effective potassium channel inhibitors with reduced side effects. We identified in the closed state a cavity formed by the S5 and S6 helices. Using docking and molecular dynamics simulation, we observe that 4-AP binds into this cavity. The binding site formed by residues L399,

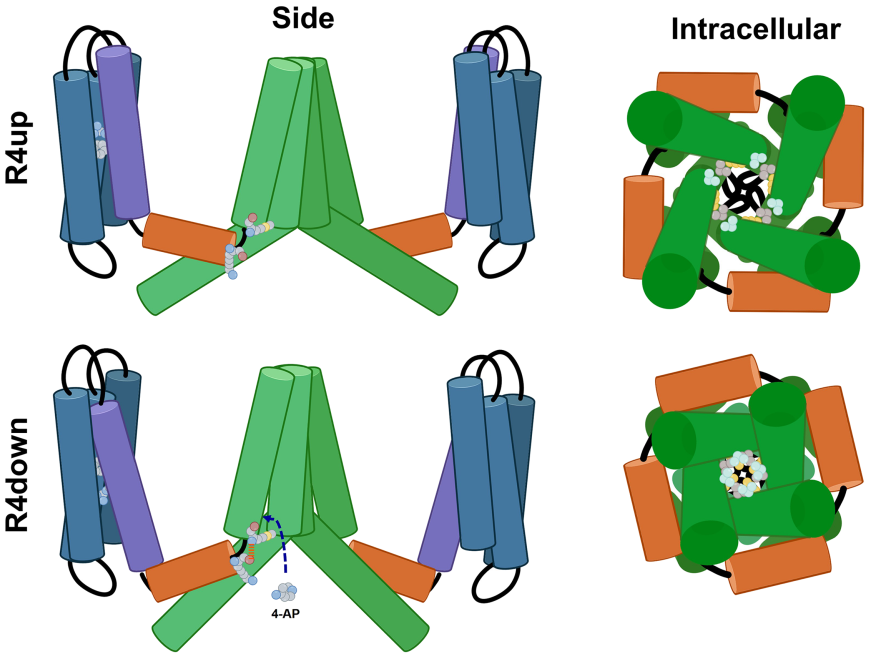

FIGURE 5 Proposed gating mechanism and 4-aminopyridine binding for the Shaker channel family. Top (R4up) Shaker channel in the active open state, where the VSD S4 segments (*purple*) are in the "R4up" conformation, and the channel pore is open (*green*). Bottom (R4down): channel in its partially activated closed state, where the VSD S4 segments are in the "R4down" position. The downward movement of the S4 segments triggers a downward and radial displacement of the S4-S5 linker (*orange*) that produces a contraction and rotation of the S6 C-terminal helices, closing the pore and allowing for the binding of 4-AP. Side view (*left*) shows two opposed subunits from a view parallel to the membrane, residues F290, R377 (R4) in the VSD and K390 and M393 in the S4-S5 linker hinge are shown. Intracellular view (*right*) shows S4-S5 linker (*orange*), S5 and S6 (*green*) from the intracellular side. Residues I470 (*yellow*), V474 (*gray*), and V478 (*pale blue*) are shown. Created with BioRender.com.

I400, L403 in the S5 and V467, L468, T469, L472, P473 in the S6 is consistent with previous reports of 4-AP affinity transplantation between Kv2.1 and Kv3.1 and point mutation analysis (34,49).

In the presence of 4-AP, the last gating component is absent in the ILT mutant (12), indicating that 4-AP, like the ILT mutations, prevents the final transition of the voltage sensor (30,50). By targeting this transition, 4-AP effectively prevents the channel from reaching the open state, thereby inhibiting its function. However, 4-AP inhibition requires channel opening (30,31). To explain these results, it has been proposed that 4-AP acts either by binding to and stabilizing the closed state; or as an open pore blocker that destabilizes the open conformation (30,31). Although we cannot rule out binding to the open pore completely, our results strongly support that 4-AP acts by stabilization of the closed state rather than as a pore blocker. We believe that, due to the coupling between VSD and PD, when the R4down state is visited there is a significant portion of open channels, thus preventing us from distinguish between binding to the open channel and binding to R4down closed state. Interestingly, 4-AP inhibition is reduced under large depolarizing pulses (31). This voltage-dependent relief of inhibition supports a mechanism in which 4-AP stabilizes the closed conformation of the channel by preventing the final translocation of R4 necessary for channel opening. Since 4-AP does not affect the rest of the gating current components and does not bind at hyperpolarized potentials, this binding pocket seems to be occluded when the channel is in more deeply closed states, like when R1-R3 are intracellularly exposed. Based on this, we propose a pot-and-lid model in which the binding site for 4-AP (the pot) exists in the closed state; however, accessibility to the binding site is prevented by a steric occlusion (the lid). In the closed states where gating charges R1-R3 are exposed to the intracellular side, the lid closes over the pot preventing 4-AP from binding. In the R4-down conformation, the lid is open, allowing 4-AP to enter the pot and bind. Upon channel opening, the pot collapses, destroying the binding site and, thus, requiring 4-AP to unbind before the channel can open. This provides a mechanistic basis for how strong depolarizations can relieve 4-AP inhibition by shifting the channel equilibrium toward the open state. Once 4-AP is bound, transitioning the channel into deeper closed states may allow the lid to close again, effectively trapping 4-AP inside the pot. We propose that this model accounts for the effects of 4-AP; however, further experiments will be required to determine its validity.

In conclusion, using AF2 we have found a model for the closed state of the pore domain of the Shaker channel that provides a mechanism for the voltage sensor coupling to the pore, which gives a molecular interpretation to the foot in the door effect of QA blockers, and unravels the site of action of the Kv channel inhibitor 4-AP.

MATERIALS AND METHODS

Conformational sampling using AlphaFold and analysis

We used AF2 (16) within the ColabFold (v1.5.2) (51) implementation to predict protein structures locally and using the online notebooks. MSAs were generated using the Mmseqs2 (52) online server integration found in ColabFold and saved for later reuse. MSA subsampling was implemented using different “max_seq” and “max_extra_seq” parameters.

For reproducibility we list all the parameters used for modeling:

	VSD MSA subsampling	Full-channel MSA subsampling	Default (R4up open)
Model	multimer v3	multimer v3	multimer v3
Number of recycles	1	1	3
Pair mode	unpaired_ paired	unpaired_paired	unpaired_paired
Use cluster profile	false	false	true
pairing_strategy	greedy	greedy	greedy
Use dropout	false	false	false
Random seed	0	0	0
Number of seeds	128,1024[a]	128,400[a]	16
Use mlm	true	True	true
Use template	no	yes[b]	yes
Models used	1,2,3,4,5	2	1,2,3,4,5
max_seq	variable	variable	508
max_extra_seq	variable	variable	2048

[a]Lower number of seeds was used for MSA subsampling parameter exploration.
[b]Except for no template case.

For modeling the monomeric VSD we used residues 228–382 of the *Drosophila melanogaster* Shaker channel (UniProt: P08510). For calculating the R4 displacement we calculated the projection of F290 and R4 α carbon to a vector defined by the S2 helix (residues 279–301) and then obtaining the distance of these two points along the S2 helix vector, the displacement was calculated relative to F290 position.

For modeling the full tetrameric channel, we used residues 215–495. During an initial run using the WT sequence we found that the selectivity filter region tends to collapse or have distortions due to the subsampling procedure. When using the sequence with the W434F mutation these distortions where minimized, and the analysis was performed using this sequence.

The area of the pore at position V474 was obtained by calculating the quadrilateral area formed by the β carbons.

Kernel density estimate plots were added to the margins of the joint plots to show the probability density distribution for each variable independently. These kernel density estimate plots were created using Seaborn’s kdeplot function with a bandwidth adjustment factor (bw_adjust = 0.2) to ensure smooth, accurate representation.

Pore radius analysis was performed using the HOLE algorithm implemented in the PoreAnalyser web service, which is based on the PoreAnalyser Python package (https://github.com/Dseiferth/PoreAnalyser2) (53,54). The protein structure was first aligned with its largest principal component along the z axis. The pore-finding algorithm was then applied using a spherical probe particle, and we used an end radius of 15 Å. Pore profiles were generated plotting the pore radius against the position along the z axis. Molecular images were prepared using ChimeraX (55).

Site-directed mutagenesis and electrophysiological recordings

Xenopus laevis ovaries were obtained from Xenopus 1 (Dexter, MI). The follicular membrane was digested by collagenase 2 mg/mL supplemented with bovine serum albumin 1 mg/mL. Oocytes were kept at 12 or 18°C in SOS solution containing: 96 mM NaCl, 2 mM KCl, 1 mM $MgCl_2$, 1.8 mM $CaCl_2$, 10 mM HEPES (pH 7.4) (NaOH) supplemented with gentamicin (50 mg/mL).

We used clones from the Shaker zH4 K^+ channel with removed N-type inactivation (IR, Δ6–46) in the pBSTA vector (56). Mutations were performed using Quikchange site-directed mutagenesis and cRNA was transcribed from linearized cDNA, using a T7 RNA kit. cRNA was injected in defolliculated oocytes (stage V-VI) and incubated in SOS solution at 18 or 12°C. After 1–4 days currents were recorded using the cut-open voltage-clamp method (57). Voltage-sensing pipettes were pulled using a horizontal puller (P-87 Model, Sutter Instruments, Novato, CA), and the resistance ranged between 0.2 and 0.5 MΩ. Data were filtered online at 20–50 kHz using a built-in low-pass four-pole Bessel filter in the voltage-clamp amplifier (CA-1B, Dagan, Minneapolis, MN) sampled at 1 MHz, digitized at 16-bits, and digitally filtered at Nyquist frequency (USB-1604; Measurement Computing, Norton, MA) using Gpatch64M (in-house software). An in-house software (Analysis) was used to acquire and analyze the data. External solution for ionic recording was composed of 12 mM K-methanosulfonate (MES), 108 mM *N*-methyl D-glucamine (NMG)-MES, 2 mM Ca-MES, 10 mM HEPES (pH 7.4), and internal solution by 120 mM K-MES, 2 mM EGTA, 10 mM HEPES (pH 7.4). External solution for gating currents recording was composed of 120 mM NMG-MES, 2 mM Ba-MES, 10 mM HEPES (pH 7.4) and internal solution by 120 mM NMG-MES, 2 mM EGTA, 10 mM HEPES (pH 7.4). 4-AP was diluted with external solution from a 200 mM stock to obtain the adequate concentration.

Electrophysiology data analysis

The G-V curves were measured from the tail currents after a voltage protocol and fitted using a two-state model given by equation:

$$G(V) = \frac{1}{1 + \exp\left(\frac{zF}{RT}\left(V - V_{1/2}\right)\right)} \quad (1)$$

where z is the apparent charge expressed in units of elementary charge (e_0), V is the voltage, and $V_{1/2}$ is the voltage of half-maximal conductance. R, T, and F have their usual meanings.

For the analysis of the Q-V curves we used two different approaches:

1) A three-state model fitting is given by the following equation (58):

$$Q(V) = N\frac{z_2 + z_1\left(1 + \left(\exp\frac{z_2F}{RT}\,(V_2 - V)\right)\right)}{1 + \exp\left(\frac{z_2F}{RT}\,(V_2 - V)\right)\left(1 + \exp\left(\frac{z_1F}{RT}\,(V_1 - V)\right)\right)} \quad (2)$$

where *N*, *z1*, *z2*, *V1*, and *V2* are the number of channels, the charges associated, and equilibrium voltages for the first and second transition, respectively.

2) A two-state model fitting equivalent to the one in Eq. 1 for the individual components in the case of ILT and ILT-393A mutants.

Molecular docking and analysis

The closed-state model of the Shaker channel (R4down model) was used as the receptor for docking analysis. The 3D structure of 4-AP was obtained from the PubChem database (CID: 1727). Both receptor and ligand were prepared using AutoDock Tools and converted to PDBQT format. Molecular docking was performed using AutoDock Vina 1.1.2 (32,33). Initially the grid box was defined to encompass the entire pore domain; after identification of the binding pocket, docking was repeated against a constrained volume encompassing a single subunit. Docking parameters included exhaustiveness set to 8 and number of output poses set to 9. Docking results were analyzed based on binding energy, with the lowest energy pose considered most favorable.

Molecular dynamics simulations and analysis

Molecular dynamics simulations were prepared using the CHARMM-GUI web interface (59) to generate input files compatible with OpenMM (60). The initial model was processed to add missing atoms and assign protonation states at pH 7.0. The protein was embedded in a preequilibrated POPC lipid bilayer, solvated with TIP3P water molecules, and neutralized with K^+ and Cl^- ions to achieve a physiological salt concentration of 0.15 M.

Energy minimization was performed for 5000 steps using a steepest descent algorithm while applying positional restraints to protein heavy atoms and lipid headgroups. The system was then equilibrated following the standard CHARMM-GUI protocol, consisting of six sequential stages with progressively decreasing positional restraints. Equilibration began with 250 ps under constant volume (NVT) conditions at 303.15 K, followed by five constant pressure (NPT) steps totaling ~2 ns. During equilibration, positional restraints on the protein backbone, side chains, and lipid headgroups were reduced stepwise from 10 to 0.1 kcal/mol/$Å^2$, allowing the system to adapt gradually to simulation conditions. Production molecular dynamics simulations were carried out in the NPT ensemble at 303.15 K and 1 atm using OpenMM. The CHARMM36m force field was used for the protein and lipids, with TIP3P water parameters (61–63). Atomic coordinates were saved every 10 ps for subsequent analysis.

The trajectory analysis was performed using MDAnalysis (v1.0.0) and NumPy in Python. The protein structure was first aligned to the reference frame (first frame) to remove translational and rotational motion. Root mean-square deviation was calculated for both the protein backbone and the drug molecule (4-AP2) relative to their initial positions. The quadrilateral area and R4 displacement in the dynamics were calculated using the same approach used for AF2 models.

Binding interactions were characterized by measuring distances between the amino group nitrogen atom (NZ) of 4-AP and the Cα atoms of key binding site residues (399, 400, 402, 468, 469, 472, 473 from chain A and residue 467 from chain D). To identify distinct binding poses, we applied principal-component analysis (PCA). To characterize the drug's orientation and position within the binding pocket, we created a multidimensional feature set capturing three geometric descriptors: the aromatic ring's normal vector, the vector pointing from the molecule's center to its pyridine nitrogen, and the drug's center of mass coordinates. The data were standardized

before PCA, and the first three principal components captured approximately 75% of the total variance.

K-means clustering was applied to the PCA-reduced data to identify distinct binding conformations. The optimal number of clusters (k = 4) was determined using the elbow method by evaluating the within-cluster sum of squares metric. For each cluster, a representative frame was selected based on the minimum Euclidean distance to the cluster centroid in the PCA space. Binding pose transitions were analyzed by tracking cluster assignments chronologically throughout the trajectory. A temporal distribution plot was created to visualize the residence time in each pose, with transitions marked by connecting lines. The occupancy percentage for each pose was calculated from frames after the 5 ns mark to exclude the initial equilibration period.

DATA AND CODE AVAILABILITY

Model obtained, data sets derived from this work and code used to generate figures is available at: https://doi.org/10.5281/zenodo.13958682.

ACKNOWLEDGMENTS

I thank Dr. Francisco Bezanilla for his mentoring, support, discussions, and constructive feedback, which significantly enhanced the quality of this work; Yichen Liu, Dr. Carlos Bassetto, and Dr. Sara T. Granados for helpful discussion of these results and Ms. Gethiely Gasparini for her technical support; Dr. Marcos Sotomayor for his insightful comments and suggestions on an earlier draft, which helped improve this manuscript; Dr. Morten Jensen for sharing the models of the Kv1.2/2.1 chimera. This work was completed in part with resources provided by the University of Chicago's Research Computing Center. The work was supported by the National Institutes of Health Award R01GM030376 (PI: Francisco Bezanilla), PEW Latin American Fellow 2019 (BPA), and Google Cloud Research Credits Grant GCP19980904 (BPA).

AUTHOR CONTRIBUTIONS

B.I.P.-A. designed and performed the research, analyzed the data, and wrote the manuscript.

DECLARATION OF INTERESTS

The author declares no competing interests.

SUPPORTING MATERIAL

Supporting material can be found online at https://doi.org/10.1016/j.bpj.2025.06.029.

REFERENCES

1. González, C., D. Baez-Nieto, …, R. Latorre. 2012. K+ channels: Function-structural overview. *Compr. Physiol.* 2:2087–2149.
2. Hille, B. 2001. Ion Channels of Excitable Membranes (3rd Edition), 3rd ed. Sinauer Associates Inc, pp. 2001–2007.
3. Wulff, H., N. A. Castle, and L. A. Pardo. 2009. Voltage-gated potassium channels as therapeutic targets. *Nat. Rev. Drug Discov.* 8:982–1001.
4. Bean, B. P. 2007. The action potential in mammalian central neurons. *Nat. Rev. Neurosci.* 8:451–465.
5. Bezanilla, F. 2008. How membrane proteins sense voltage. *Nat. Rev. Mol. Cell Biol.* 9:323–332.
6. Blunck, R., and Z. Batulan. 2012. Mechanism of electromechanical coupling in voltage-gated potassium channels. *Front. Pharmacol.* 3:166.
7. Seoh, S. A., D. Sigg, …, F. Bezanilla. 1996. Voltage-sensing residues in the S2 and S4 segments of the Shaker K+ channel. *Neuron.* 16:1159–1167.
8. Aggarwal, S. K., and R. MacKinnon. 1996. Contribution of the S4 segment to gating charge in the Shaker K+ channel. *Neuron.* 16:1169–1177.
9. Starace, D. M., and F. Bezanilla. 2004. A proton pore in a potassium channel voltage sensor reveals a focused electric field. *Nature.* 427:548–553.
10. Starace, D. M., and F. Bezanilla. 2001. Histidine scanning mutagenesis of basic residues of the S4 segment of the shaker k+ channel. *J. Gen. Physiol.* 117:469–490.
11. Tao, X., A. Lee, …, R. MacKinnon. 2010. A Gating Charge Transfer Center in Voltage Sensors. *Science.* 328:67–73.
12. Lacroix, J. J., and F. Bezanilla. 2011. Control of a final gating charge transition by a hydrophobic residue in the S2 segment of a K+ channel voltage sensor. *Proc. Natl. Acad. Sci. USA.* 108:6444–6449.
13. Nashmi, R., and M. G. Fehlings. 2001. Mechanisms of axonal dysfunction after spinal cord injury: With an emphasis on the role of voltage-gated potassium channels. *Brain Res. Rev.* 38:165–191.
14. Goodman, A. D., T. R. Brown; …, Fampridine MS-F203 Investigators. 2009. Sustained-release oral fampridine in multiple sclerosis: a randomised, double-blind, controlled trial. *Lancet.* 373:732–738.
15. Korenke, A. R., M. P. Rivey, and D. R. Allington. 2008. Sustained-release fampridine for symptomatic treatment of multiple sclerosis. *Ann. Pharmacother.* 42:1458–1465.
16. Jumper, J., R. Evans, …, D. Hassabis. 2021. Highly accurate protein structure prediction with AlphaFold. *Nature.* 596:583–589.
17. Evans, R., M. O'Neill, …, D. Hassabis. 2022. Protein complex prediction with AlphaFold-Multimer. Preprint at bioRxiv. https://doi.org/10.1101/2021.10.04.463034.
18. Baek, M., F. DiMaio, …, D. Baker. 2021. Accurate prediction of protein structures and interactions using a three-track neural network. *Science.* 373:871–876.
19. del Alamo, D., D. Sala, …, J. Meiler. 2022. Sampling alternative conformational states of transporters and receptors with AlphaFold2. *eLife.* 11:e75751.
20. Heo, L., and M. Feig. 2022. Multi-state modeling of G-protein coupled receptors at experimental accuracy. *Proteins.* 90:1873–1885.
21. Monteiro da Silva, G., J. Y. Cui, …, B. M. Rubenstein. 2024. High-throughput prediction of protein conformational distributions with subsampled AlphaFold2. *Nat. Commun.* 15:2464.
22. Tan, X. F., C. Bae, …, K. J. Swartz. 2022. Structure of the Shaker Kv channel and mechanism of slow C-type inactivation. *Sci. Adv.* 8:eabm7814.
23. Lacroix, J. J., H. C. Hyde, …, F. Bezanilla. 2014. Moving gating charges through the gating pore in a Kv channel voltage sensor. *Proc. Natl. Acad. Sci. USA.* 111:E1950–E1959.
24. Jumper, J., R. Evans, …, D. Hassabis. 2021. Applying and improving AlphaFold at CASP14. *Proteins.* 89:1711–1721.
25. Liu, Y., M. Holmgren, …, G. Yellen. 1997. Gated access to the pore of a voltage-dependent K+ channel. *Neuron.* 19:175–184.
26. Ye, W., H. Zhao, …, C.-H. Lee. 2022. Activation and closed-state inactivation mechanisms of the human voltage-gated KV4 channel complexes. *Mol. Cell.* 82:2427–2442.e4.
27. Jensen, M. Ø., V. Jogini, …, D. E. Shaw. 2012. Mechanism of Voltage Gating in Potassium Channels. *Science.* 336:229–233.
28. Perozo, E., R. MacKinnon, …, E. Stefani. 1993. Gating currents from a nonconducting mutant reveal open-closed conformations in Shaker K+ channels. *Neuron.* 11:353–358.

29. Ledwell, J. L., and R. W. Aldrich. 1999. Mutations in the S4 region isolate the final voltage-dependent cooperative step in potassium channel activation. *J. Gen. Physiol.* 113:389–414.

30. Armstrong, C. M., and A. Loboda. 2001. A Model for 4-Aminopyridine Action on K Channels: Similarities to Tetraethylammonium Ion Action. *Biophys. J.* 81:895–904.

31. Mccormack, K., W. J. Joiner, and S. H. Heinemann. 1994. A Characterization of the Activating Structural Rearrangements in Voltage-Dependent Shaker K+ Channels. *Neuron.* 12:301–315.

32. Eberhardt, J., D. Santos-Martins, …, S. Forli. 2021. AutoDock Vina 1.2.0: New Docking Methods, Expanded Force Field, and Python Bindings. *J. Chem. Inf. Model.* 61:3891–3898.

33. Trott, O., and A. J. Olson. 2010. AutoDock Vina: Improving the speed and accuracy of docking with a new scoring function, efficient optimization, and multithreading. *J. Comput. Chem.* 31:455–461.

34. Kirsch, G. E., C. C. Shieh, …, A. M. Brown. 1993. Segmental exchanges define 4-aminopyridine binding and the inner mouth of K+ pores. *Neuron.* 11:503–512.

35. Mandala, V. S., and R. MacKinnon. 2022. Voltage-sensor movements in the Eag Kv channel under an applied electric field. *Proc. Natl. Acad. Sci. USA.* 119:e2214151119.

36. Wisedchaisri, G., L. Tonggu, …, W. A. Catterall. 2019. Resting-State Structure and Gating Mechanism of a Voltage-Gated Sodium Channel. *Cell.* 178:993–1003.e12.

37. Guo, J., W. Zeng, …, Y. Jiang. 2016. Structure of the voltage-gated two-pore channel TPC1 from Arabidopsis thaliana. *Nature.* 531:196–201.

38. Barros, F., L. A. Pardo, …, P. De la Peña. 2019. New Structures and Gating of Voltage-Dependent Potassium (Kv) Channels and Their Relatives: A Multi-Domain and Dynamic Question. *Int. J. Mol. Sci.* 20:248.

39. Labro, A. J., A. L. Raes, …, D. J. Snyders. 2008. Kv channel gating requires a compatible S4-S5 linker and bottom part of S6, constrained by non-interacting residues. *J. Gen. Physiol.* 132:667–680.

40. Haddad, G. A., and R. Blunck. 2011. Mode shift of the voltage sensors in Shaker K+ channels is caused by energetic coupling to the pore domain. *J. Gen. Physiol.* 137:455–472.

41. Lu, Z., A. M. Klem, and Y. Ramu. 2002. Coupling between voltage sensors and activation gate in voltage-gated K+ channels. *J. Gen. Physiol.* 120:663–676.

42. Lu, Z., A. M. Klem, and Y. Ramu. 2001. Ion conduction pore is conserved among potassium channels. *Nature.* 413:809–813.

43. Zhou, M., J. H. Morais-Cabral, …, R. MacKinnon. 2001. Potassium channel receptor site for the inactivation gate and quaternary amine inhibitors. *Nature.* 411:657–661.

44. Yellen, G., M. E. Jurman, …, R. MacKinnon. 1991. Mutations Affecting Internal TEA Blockade Identify the Probable Pore-Forming Region of a K + Channel. *Science.* 251:939–942.

45. Yeh, J. Z., and C. M. Armstrong. 1978. Immobilisation of gating charge by a substance that simulates inactivation. *Nature.* 273:387–389.

46. Armstrong, C. M. 1971. Interaction of tetraethylammonium ion derivatives with the potassium channels of giant axons. *J. Gen. Physiol.* 58:413–437.

47. Holmgren, M., P. L. Smith, and G. Yellen. 1997. Trapping of organic blockers by closing of voltage-dependent K+ channels: Evidence for a trap door mechanism of activation gating. *J. Gen. Physiol.* 109:527–535.

48. Melishchuk, A., and C. M. Armstrong. 2001. Mechanism Underlying Slow Kinetics of the OFF Gating Current in Shaker Potassium Channel. *Biophys. J.* 80:2167–2175.

49. Shieh, C. C., and G. E. Kirsch. 1994. Mutational analysis of ion conduction and drug binding sites in the inner mouth of voltage-gated K+ channels. *Biophys. J.* 67:2316–2325.

50. Loboda, A., and C. M. Armstrong. 2001. Resolving the gating charge movement associated with late transitions in K channel activation. *Biophys. J.* 81:905–916.

51. Mirdita, M., K. Schütze, …, M. Steinegger. 2022. ColabFold: making protein folding accessible to all. *Nat. Methods.* 19:679–682.

52. Mirdita, M., M. Steinegger, and J. Söding. 2019. MMseqs2 desktop and local web server app for fast, interactive sequence searches. *Bioinformatics.* 35:2856–2858.

53. Smart, O. S., J. G. Neduvelil, …, M. S. Sansom. 1996. HOLE: A program for the analysis of the pore dimensions of ion channel structural models. *J. Mol. Graph.* 14:354-60–376.

54. Seiferth, D., and P. C. Biggin. 2024. Exploring the influence of pore shape on conductance and permeation. *Biophys. J.* 123:3107–3119.

55. Pettersen, E. F., T. D. Goddard, …, T. E. Ferrin. 2021. UCSF ChimeraX: Structure visualization for researchers, educators, and developers. *Protein Sci.* 30:70–82.

56. Hoshi, T., W. N. Zagotta, and R. W. Aldrich. 1990. Biophysical and Molecular Mechanisms of Shaker Potassium Channel Inactivation. *Science.* 250:533–538.

57. Stefani, E., and F. Bezanilla. 1998. Cut-open oocyte voltage-clamp technique. *In* Methods in Enzymology. Department of Anesthesiology. P. Michael Conn, ed University of California, pp. 300–318.

58. Lacroix, J. J., S. A. Pless, …, F. Bezanilla. 2012. Intermediate state trapping of a voltage sensor. *J. Gen. Physiol.* 140:635–652.

59. Jo, S., T. Kim, …, W. Im. 2008. CHARMM-GUI: A web-based graphical user interface for CHARMM. *J. Comput. Chem.* 29:1859–1865.

60. Eastman, P., R. Galvelis, …, T. E. Markland. 2024. OpenMM 8: Molecular Dynamics Simulation with Machine Learning Potentials. *J. Phys. Chem. B.* 128:109–116.

61. Huang, J., S. Rauscher, …, A. D. MacKerell. 2017. CHARMM36m: an improved force field for folded and intrinsically disordered proteins. *Nat. Methods.* 14:71–73.

62. Jorgensen, W. L., J. Chandrasekhar, …, M. L. Klein. 1983. Comparison of simple potential functions for simulating liquid water. *J. Chem. Phys.* 79:926–935.

63. Klauda, J. B., R. M. Venable, …, R. W. Pastor. 2010. Update of the CHARMM All-Atom Additive Force Field for Lipids: Validation on Six Lipid Types. *J. Phys. Chem. B.* 114:7830–7843.

Biophysical Journal, Volume *124*

Supplemental information

Voltage gating and 4-aminopyridine inhibition in the Shaker Kv channel revealed by a closed-state model

Bernardo I. Pinto-Anwandter

Supplementary information

Voltage Gating and 4-aminopyridine Inhibition in Shaker Kv Channel Revealed by Closed-State Model

Authors

Bernardo I Pinto-Anwandter [1*]

Affiliations

1 Department of Biochemistry and Molecular Biology, University of Chicago, Chicago, IL, 60637, USA

Correspondence

* Corresponding author, correspondence to pintobi@uchicago.edu

Supplementary Figures

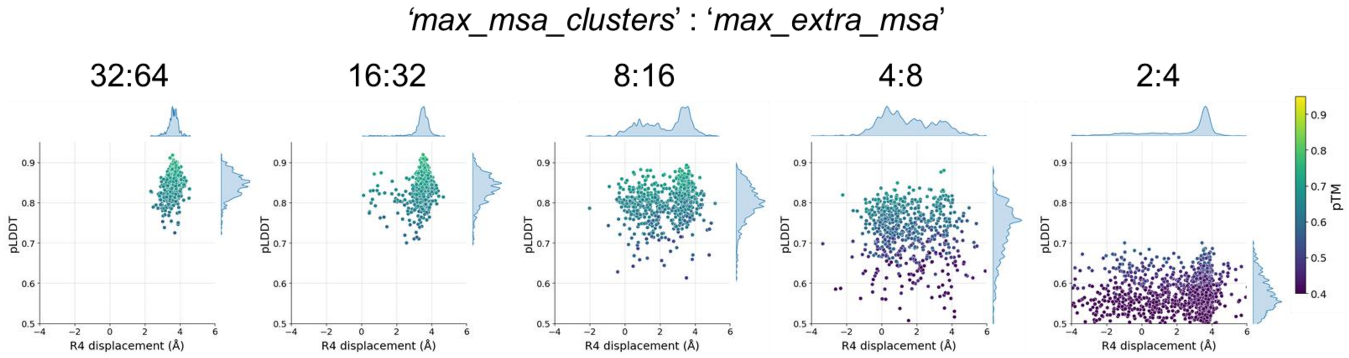

Supplementary figure 1: Conformational sampling parameter exploration for the Shaker VSD. Plot of R4 displacement vs pLDDT for AF2 generated models (600 models per plot) of Shaker VSD, using different '*max_msa_clusters*' and '*max_extra_msa*' parameters. Points colored according to pTM score. Side plots show the kernel density estimates distribution for each axis.

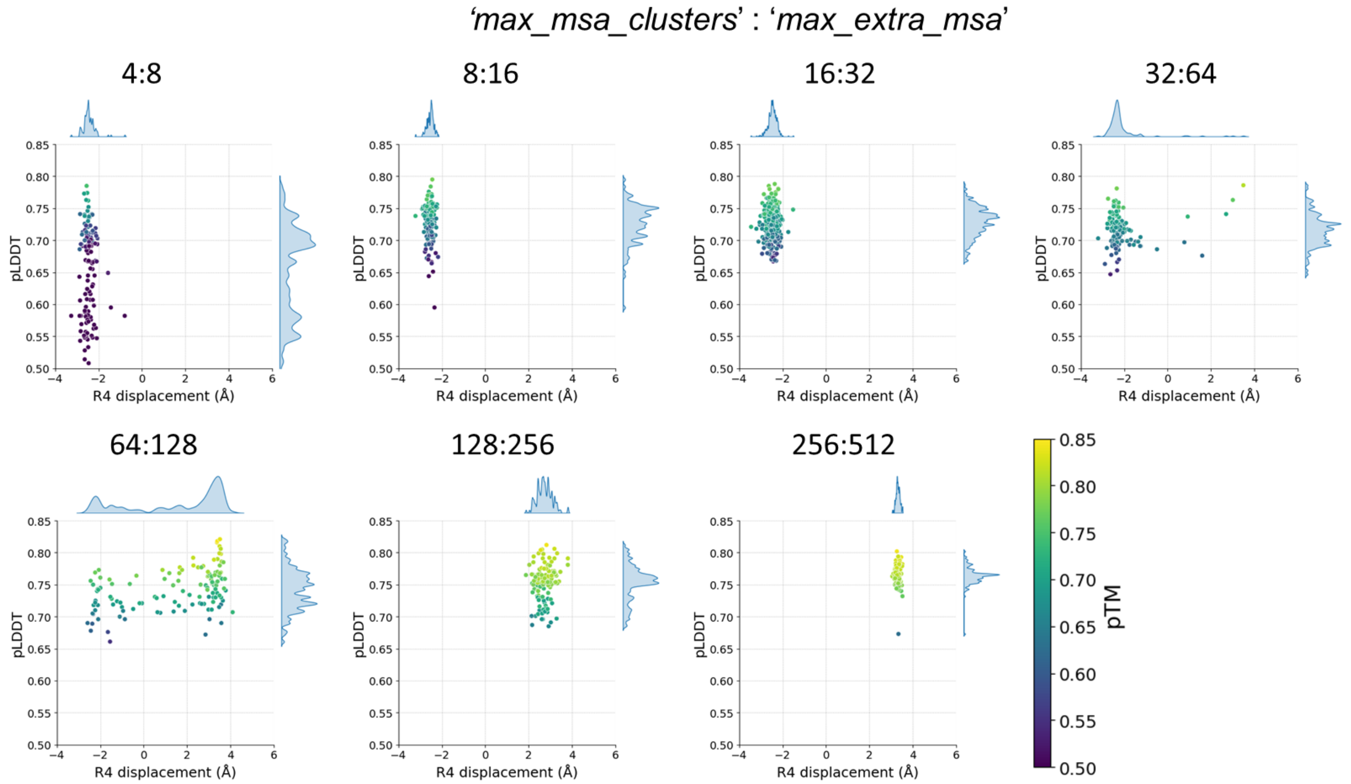

Supplementary figure 2: R4 displacement for the full channel using R4down template and different MSA subsampling parameters. Plot of R4 displacement vs pLDDT for AF2 generated models (128 models per plot) of Shaker tetrameric channel (residues 215-495) using R4down template using different MSA subsampling depth parameters. Points colored according to pTM. Side plots show the kernel density estimates distribution for each axis.

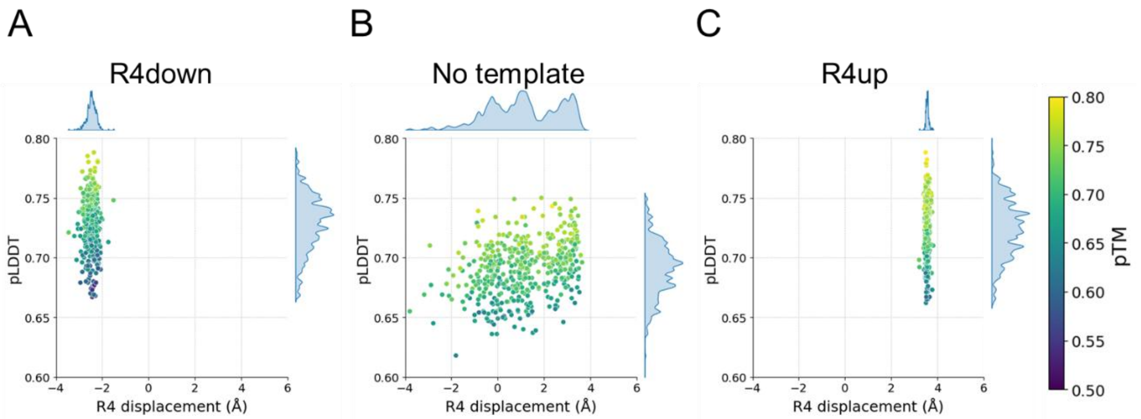

Supplementary figure 3: R4 displacement for the full channel with different VSD templates. Plot of R4 displacement vs pLDDT for AF2 generated models (400 models per plot) of Shaker tetrameric channel (residues 215-495) using R4down template (**A**), no template (**B**) or R4up template (**C**). MSA subsampling parameters used was 16:32. Points colored according to pTM. Side plots show the kernel density estimates distribution for each axis.

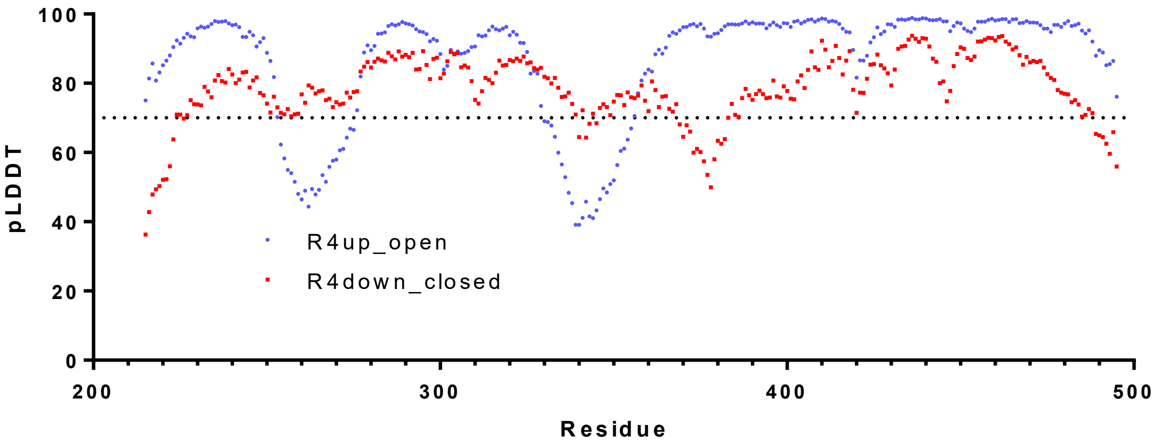

Supplementary figure 4: Per residue pLDDT metrics. Plot of the pLDDT values for each residue in the selected closed and open states. The dashed line indicates the cutoff values of 70 used to discriminate high pLDDT values.

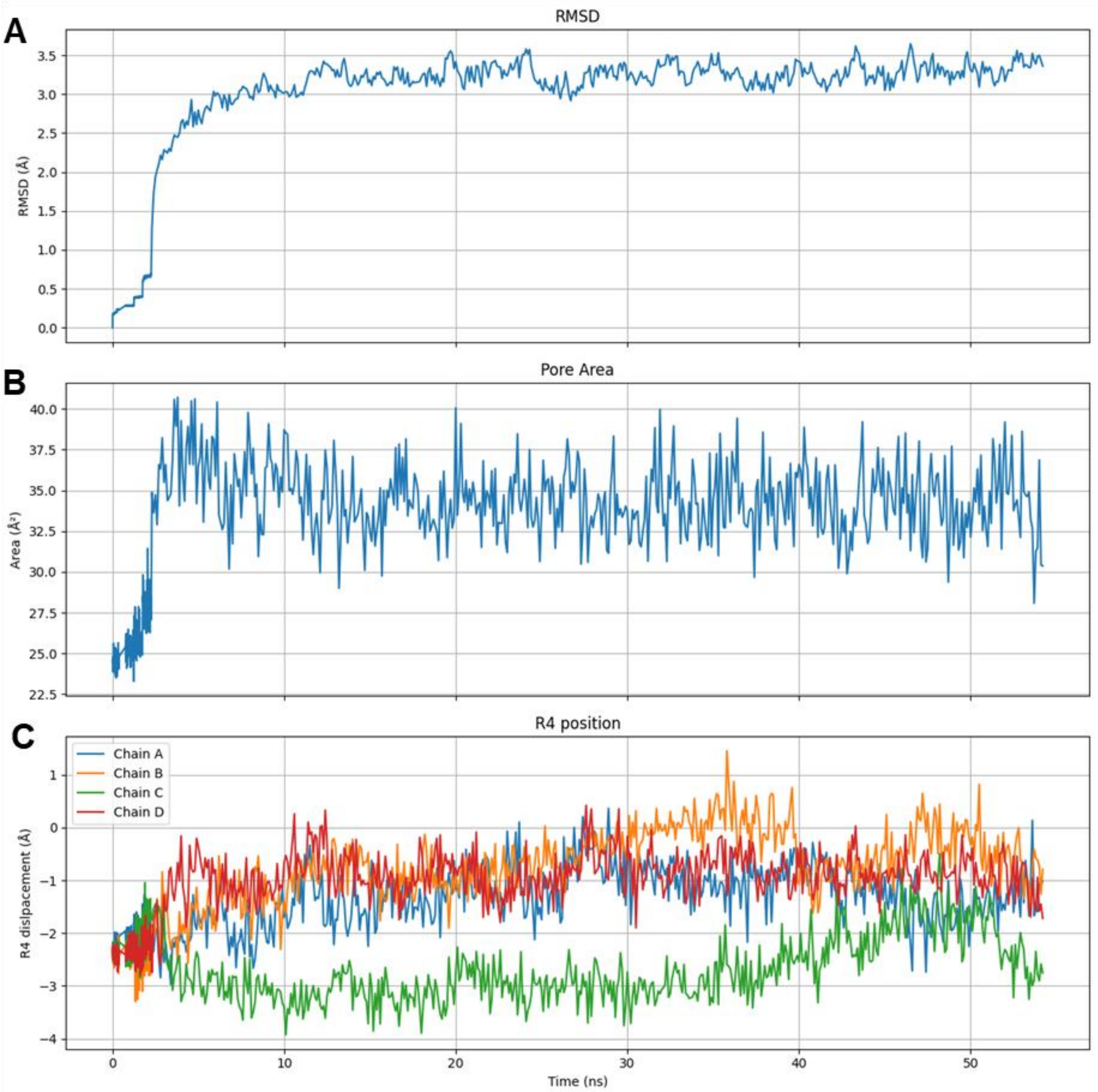

Supplementary figure 5: Analysis of molecular dynamics simulations of the closed state. **A)** Backbone RMSD, **B)** quadrilateral pore area at the level of V474 and **C**) R4 displacement for each VSD of the closed R4down model during a 55 ns MD simulation. The first 2 ns correspond to the equilibration of the system.

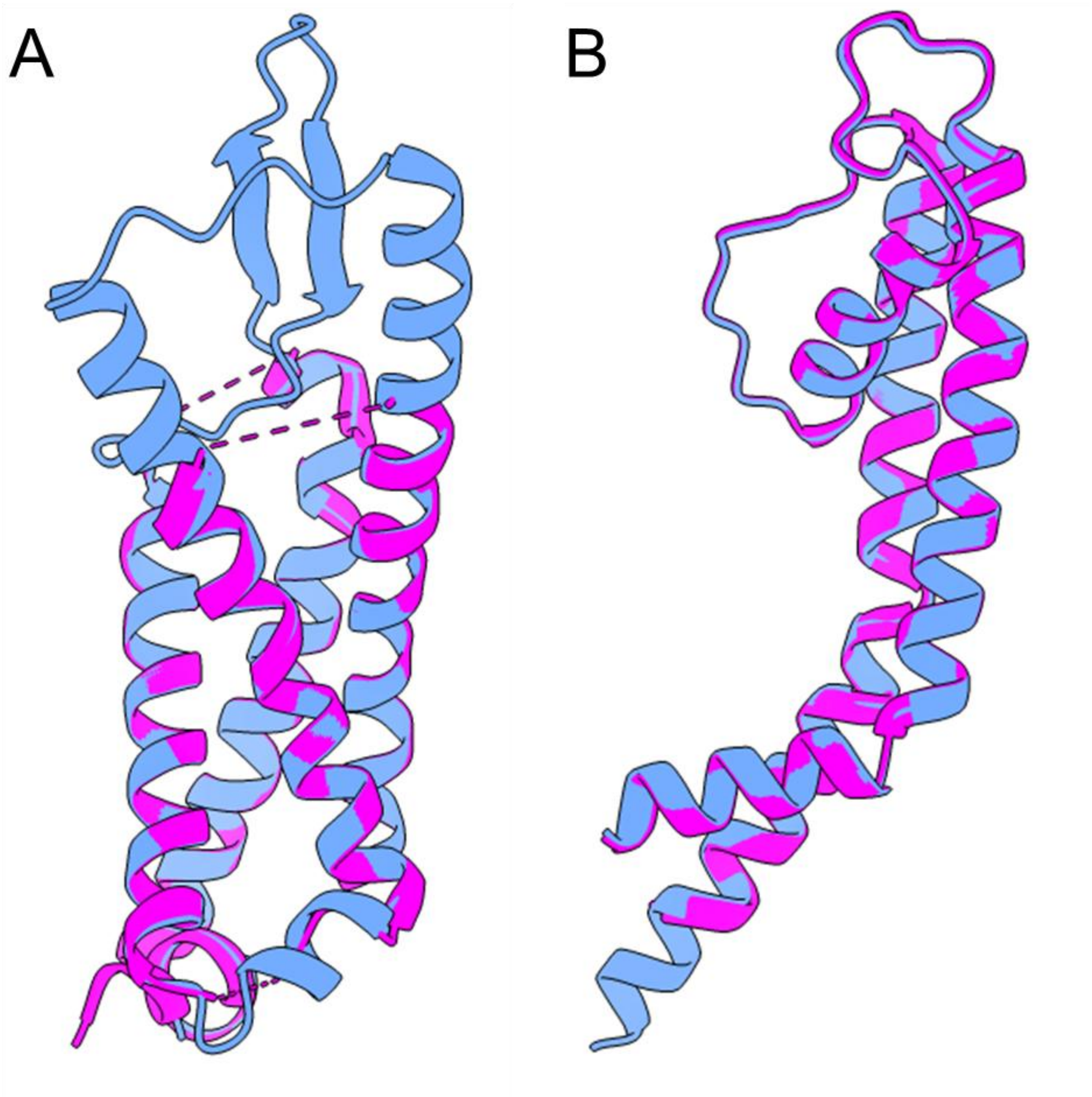

Supplementary figure 6: Comparison between R4up open model and structure of the WT channel. Aligned structures of the WT channel (PDB:7sip (22), magenta) and R4up open model (blue) for the VSD (**A**) and PD (**B**) of a single subunit (RMSD: 0.87 Å).

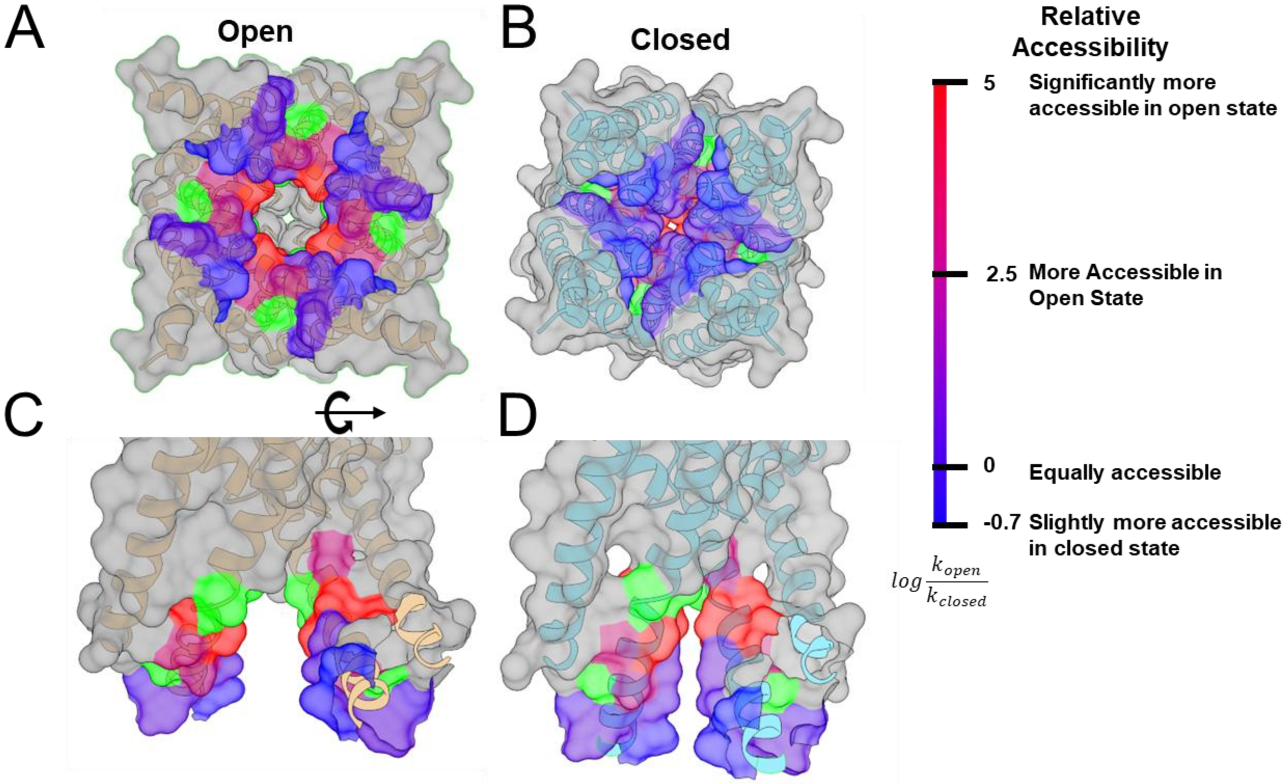

Supplementary figure 7: Mapping relative accessibility data into open and closed conformations. Surface representation of the PD of open (**A, C**) and closed (**B, D**) models colored according to experimental data of relative accessibility changes between open and closed states. Data adapted from ref (26), relative accessibility calculated as log (k_{open}/k_{closed}), when k_{closed} was unable to be measured (less than 1 M s^{-1}) value was set to 5. Green indicates residues that show no discernible effect upon application of modifying reagents. Gray indicates residues not tested.

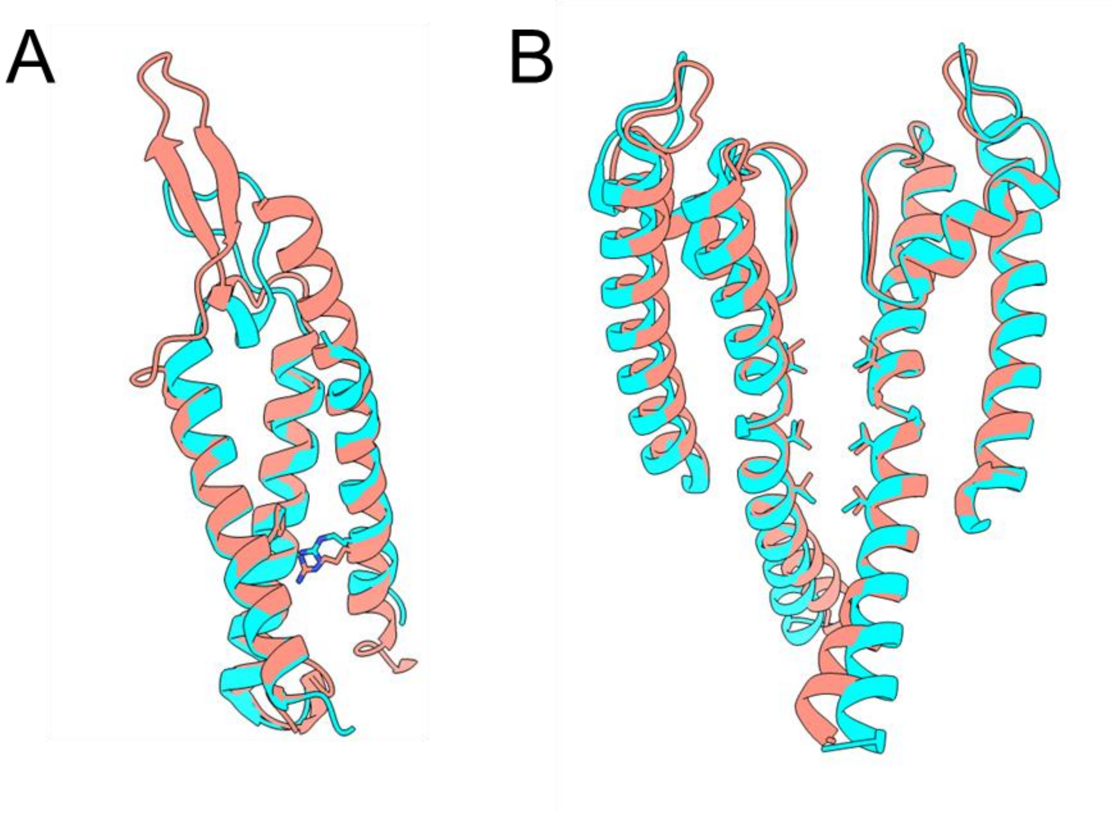

Supplementary figure 8: Comparison between R4down closed model and structure of putative resting Kv4.1 channel. Aligned structures of the putative resting Kv4.1 channel (PDB:7UKF (27), cyan) and R4up closed model (orange) for the VSD (S3 removed) of a single subunit (**A**) and PD of opposed subunits (**B**).

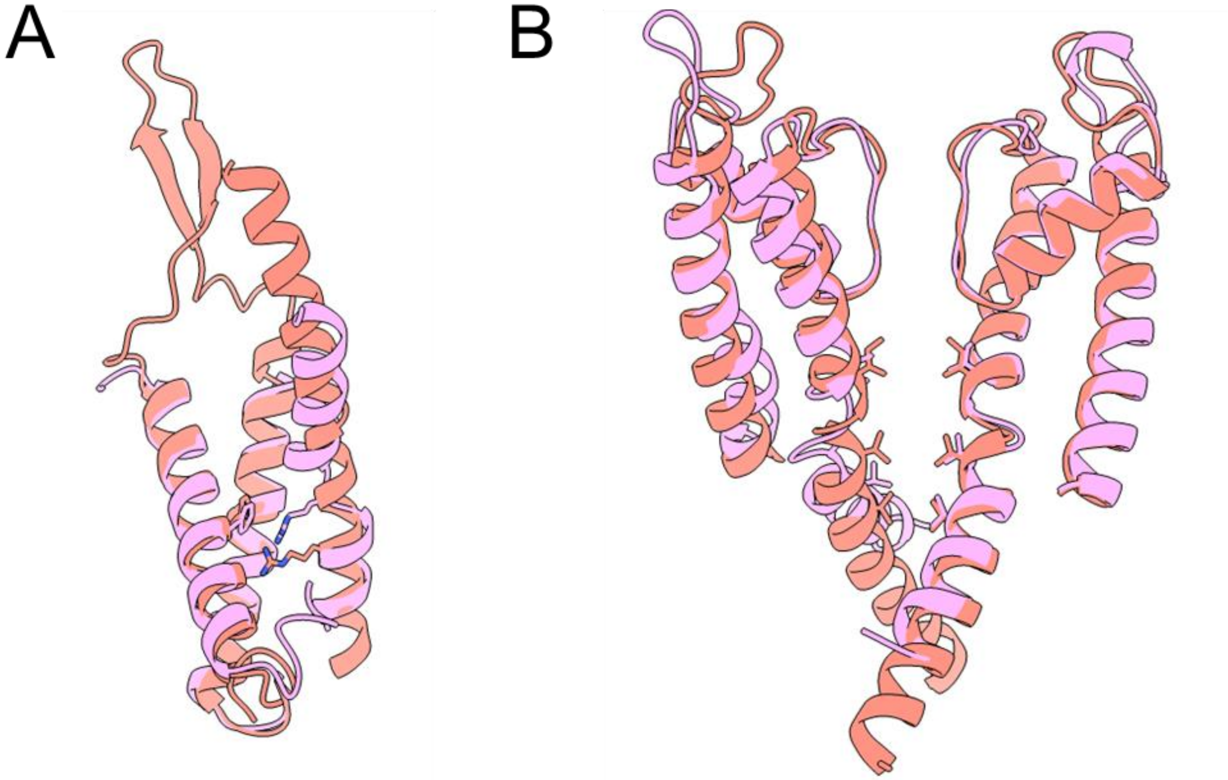

Supplementary figure 9: Comparison between R4down closed model and late deactivated state model from Jensen et al. (28)**.** Aligned late deactivated model of the Kv1.2/2.1 chimera (pink) and R4down closed model (orange) for the VSD of a single subunit (S3 removed) (**A**) and PD of opposed subunits (**B**).

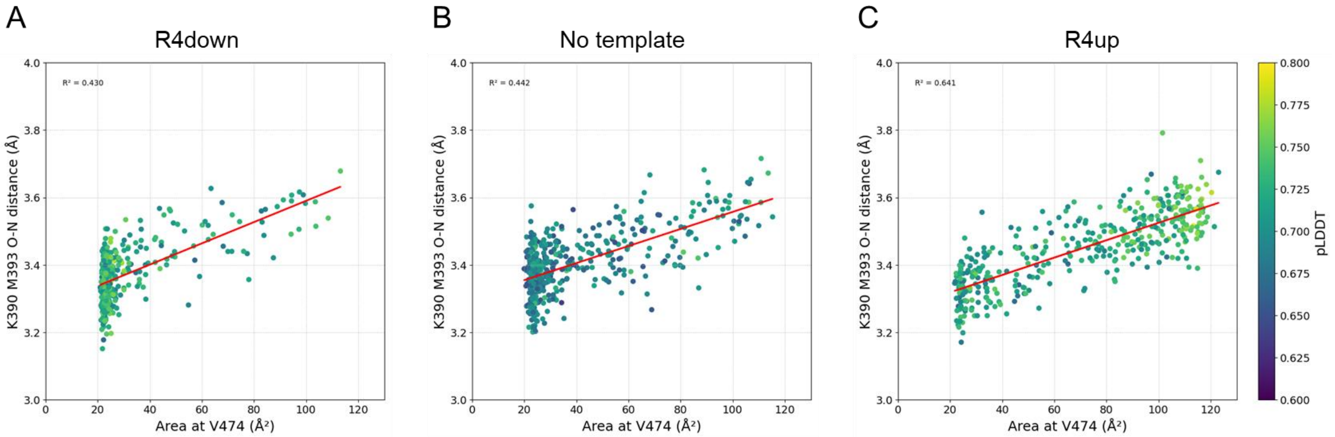

Supplementary figure 10: Relation between the area of the pore and the K390-M393 O-N distance for the full channel with different VSD templates. Plot of quadrilateral area at the level of V474 vs K390-393 O-N distance for AF2 generated models (400 models per plot) of Shaker tetrameric channel (residues 115-495) using R4down template (**A**), no template (**B**) or R4up template (**C**). MSA subsampling parameters used was 16:32. Points colored according to pLDDT. Red line shows linear fit.

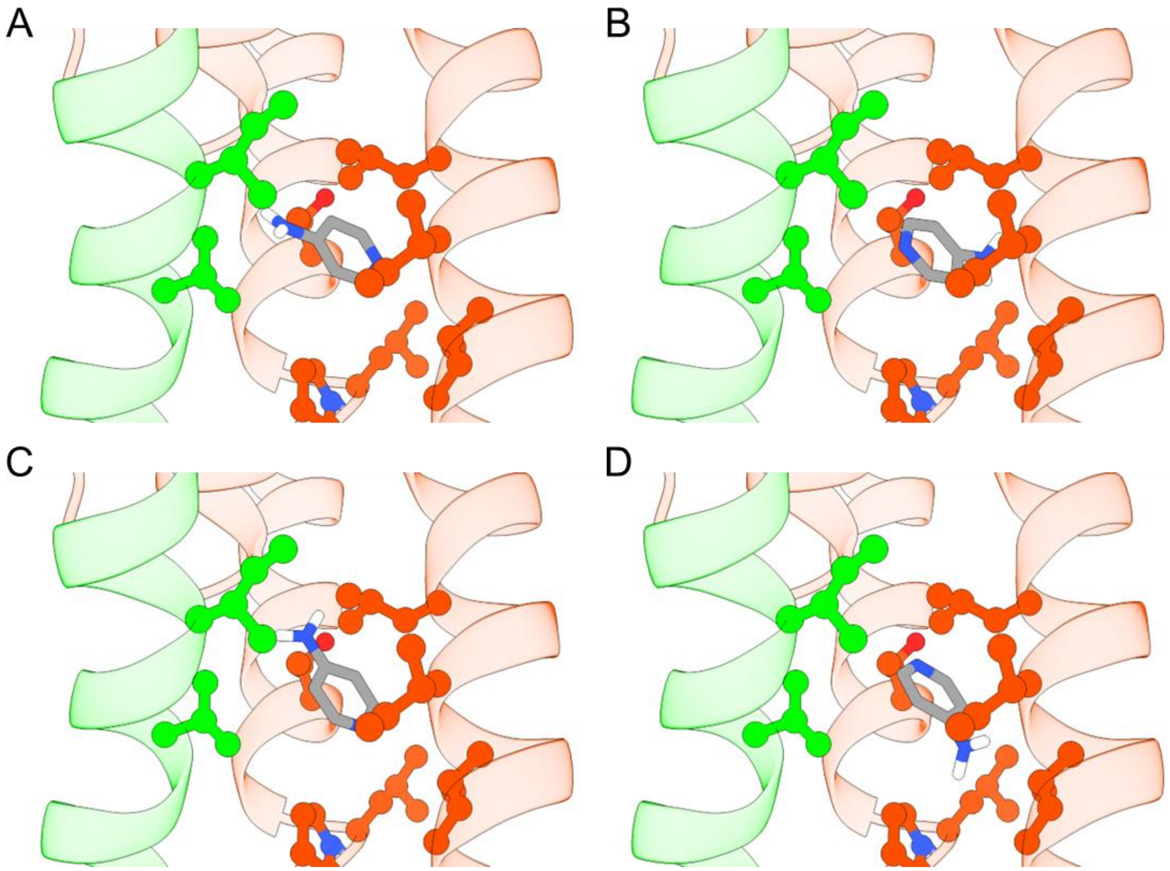

Supplementary figure 11: Docking poses and binding site of 4-AP. A-D) Representative structures of the four most favorable docking conformation identified. The protein is shown in cartoon representation with one subunit S5 and S6 in red and an adjacent subunit S5 in green, binding pocket residues are shown in stick representation. The predicted binding energies for each model are (**A**) -4.2, (**B**) -4, (**C**) -3.9 and (**D**) -3.8 kcal/mol.

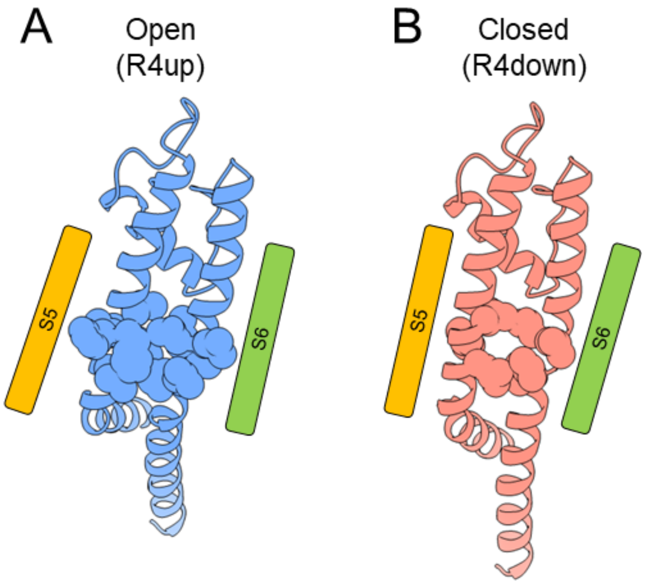

Supplementary figure 12: Formation of the 4-AP binding cavity on the closed state. Open (**A**) and closed (**B**) state models showing the PD region that forms the 4-AP binding cavity. Shown in sphere representation are the residues in S5 (398 to 403) and S6 (468 to 473) that form the cavity.

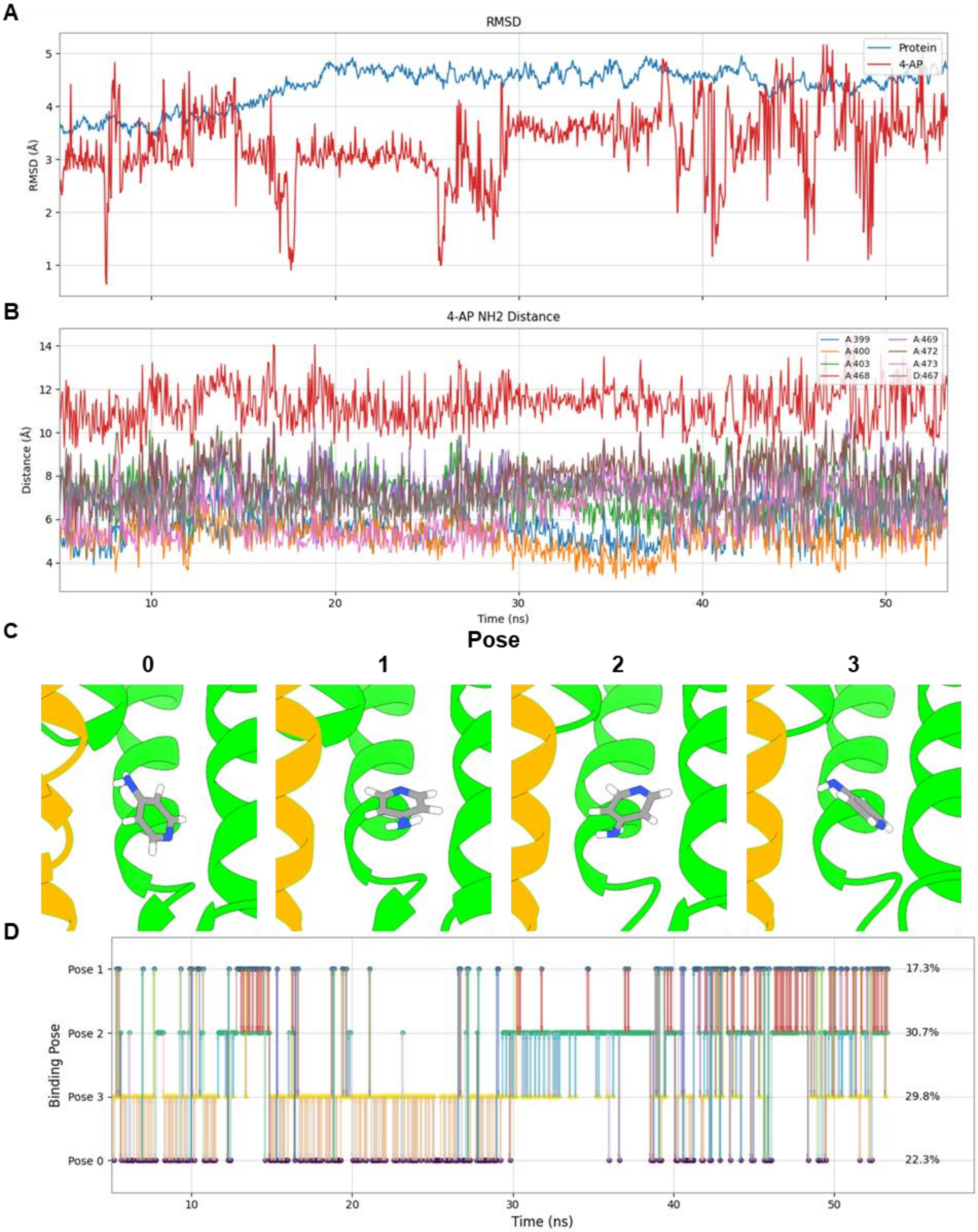

Supplementary Figure 13. Analysis of molecular dynamics simulations of the 4-AP bound state. A) Backbone RMSD of protein (blue) and 4-AP (red) over the 50 ns simulation. **B**) Time evolution of distances between the 4-AP NH_2 group and Cα atoms of the binding site. **C**) Representative structures of the four distinct binding poses (0-3) identified. The protein is shown in cartoon representation with one subunit in S5 and S6 in green and an adjacent subunit S5 in yellow, 4-AP is displayed in stick representation. D) Temporal distribution of the four binding

poses over the simulation Pose transitions are represented by vertical lines connecting the different states. Trajectories were analyzed after 5 ns.

Supplementary Table 1: GV fit parameters Shaker WT, ILT and 393 mutants

	V50 (mV)	z (e_0)
**WT**	-21.1 ± 0.5	2.6 ± 0.4
**M393A**	-26.25 ± 0.8	2.3 ± 0.7
**M393P**	-60 .1 ± 0.4	4.8 ± 0.4
**ILT**	133 ± 2	1.17 ± 0.06
**ILT-M393A**	107.3 ± 0.4	1.98 ± 0.05
**ILT-M393P**	0.4 ± 0.9	2.4 ± 0.2

*Fits were calculated using a two-state model (Eq. 1)

Supplementary Table 2: QV parameters for Shaker WT, ILT and 393 mutants

	z1 (e_0)	V1 (mV)	z2 (e_0)	V2 (mV)
**WT**	1.9 ± 0.3	-55 ± 2	3.5 ± 0.3	-34 ± 1
**M393A**	1.3 ± 0.2	-66 ± 3	3.3 ± 0.3	-46 ± 1
**M393P**	1.6 ± 0.3	-69 ± 4	4.5 ± 0.2	-72 ± 1
**ILT**	1.93 ± 0.06	-87.6 ± 0.5	1.5 ± 0.2	127 ± 4
**ILT-M393A**	1.9 ± 0.06	-92.5 ± 0.5	2.0 ± 0.1	104 ± 1
**ILT-M393P**	1 ± 0.1	-97 ± 9	0.5 ± 0.1	-76 ± 15

*Fits were calculated using a sequential three state model (Eq. 2) for WT, 393P, 393A and ILT_393P or fitting each component separately for ILT and ILT_393A (Eq. 1).
